# Tyrosinase Driven‐Intracellular Polymerization of a Porphyrin Derivative Induced Immunogenic Death of Melanoma Cells and Strengthened Photodynamic Therapy

**DOI:** 10.1002/advs.75353

**Published:** 2026-04-20

**Authors:** Mian Tang, Junteng Qiu, Yunfeng Lu, Yahui Song, Xinyu Zhou, Tianshun Duan, Xuanqi Peng, Caixia Yin, Cheng Gao, Ruibing Wang

**Affiliations:** ^1^ State Key Laboratory of Mechanism and Quality of Chinese Medicine, Institute of Chinese Medical Sciences, and MoE Frontiers Science Center for Precision Oncology University of Macau Macau SAR P. R. China; ^2^ Key Laboratory of Chemical Biology and Molecular Engineering of Ministry of Education Institute of Molecular Science Shanxi University Taiyuan P. R. China; ^3^ School of Pharmacy Shenzhen University Medical School Shenzhen University Shenzhen P. R. China

**Keywords:** immunogenic cell death, intracellular polymerization, photodynamic therapy, tyrosinase

## Abstract

Therapeutic agents usually must reach diseased tissue and then exert biological effects. While numerous targeted and stimuli‐responsive delivery systems have been developed, their therapeutic outcomes have been limited due to the lack of “precision”. In this study, we present a strategy for in situ polymerization of a porphyrin derivative within melanoma cells triggered by tyrosinase, and subsequent self‐assembly of the polymers into microstructures limiting porphyrin efflux, to achieve “precision medicine” for melanoma. The specific intracellular polymerization and subsequent self‐assembly process induced immunogenic cell death of melanoma cells and transformed immunosuppressive “cold” tumors into immunoactive “hot” tumors by polarizing macrophage into M1 type, stimulating dendritic cell maturation, and improving CD8 T cell infiltration and activation. Furthermore, the intracellularly retained polymeric porphyrin microstructures  enhanced photodynamic therapy to synergistically drive improved treatment outcomes. This in situ intracellular polymerization and self‐assembly approach provides a precise and safe alternative to conventional drug‐based cancer therapies.

## Graphical abstract

This study uses tyrosinase, which is highly expressed in melanoma, to drive porphyrins to polymerize and self‐assemble within tumor cells to form retented microstructures. This process induces immunogenic death and activates immune responses (M1 macrophages, dendritic cells, CD8^+^T cells), turning “cold” tumors into “hot” tumors. Combined with photodynamic therapy, it synergizes and enhances efficacy, providing a new strategy for precision tumor treatment.

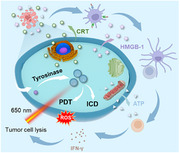

## 1 Introduction

In recent years, a plethora of nanomedicines have been developed for the diagnosis and treatment of a variety of diseases, particularly cancer [1, 2, 3]. Most of these nanomedicines rely on the enhanced permeability and retention (EPR) effect of nanoparticles to enhance drug accumulation at tumor sites [4, 5]. However, studies have shown that less than 0.0014% of the administered nanomedicines reach the lesion site [6]. The majority are trapped in the extracellular matrix, phagocytosed by tumor‐associated macrophages surrounding blood vessels, or transferred to healthy tissues, causing a burden on organs. Therefore, it is necessary to explore alternative approaches for precision cancer treatment.

Enzymes, ubiquitous in living organisms, serve as potent catalysts with high precision and efficiency [7]. Within the tumor microenvironment, multiple enzymes are overexpressed and exhibit functional diversification. For instance, alkaline phosphatase (ALP) [8], γ‐glutamyl transpeptidase (GGT) [9], and lactate dehydrogenase (LDH) [10, 11], serve as key biomarkers for the occurrence of hepatobiliary and aggressive hematological tumors and their dynamic progression. Hyaluronidase as a bi‐functional catalyst, simultaneously promotes the tumor invasion/metastasis cascade, enhances the penetration of therapeutic agents, and modulates the immune microenvironment [12, 13]. On the other hand, endogenous intracellular enzymes could be utilized for the biosynthesis of functional substances to regulate the physiological activities of cells [14, 15, 16], presenting a promising strategy for cancer regulation [17, 18, 19]. Tyrosinase is an enzyme overexpressed in melanoma cells [20, 21, 22]. It catalyzes the generation of melanin granules through the continuous oxidation of tyrosine to dopamine (o‐diphenol), followed by the oxidation of dopamine to quinone (o‐diquinone); these quinones are then encapsulated by melanosomes and transported to various locations within the cell [23, 24]. Thus, tyrosinase could be utilized as a precise catalyst for the development of precision melanoma therapy.

On the other hand, the direct synthesis of bioactive polymeric molecules within living cells represents a revolutionary “in situ synthesis” strategy. This technology leverages the endogenous cellular environment to construct a non‐natural, cell‐compatible, and functional polymer network inside the cell, possibly endowing cells with tunable properties. A key advantage lies in its exceptionally high specificity—the polymerization reaction can be programmed to be triggered exclusively by disease‐specific biological stimuli, such as enzyme overexpression [25, 26], accumulation of reducing substances [27, 28], elevated reactive oxygen species (ROS) levels [29, 30, 31, 32], or external light exposure [33, 34]. Since polymer formation is precisely confined to the specific cell's microenvironment, this method ensures high precision. Furthermore, once the polymer network is formed inside the cell, it is resistant to cellular expulsion or clearance, enabling prolonged residence and continuous function, thereby providing sustained therapeutic effects [35].

**SCHEME 1:**
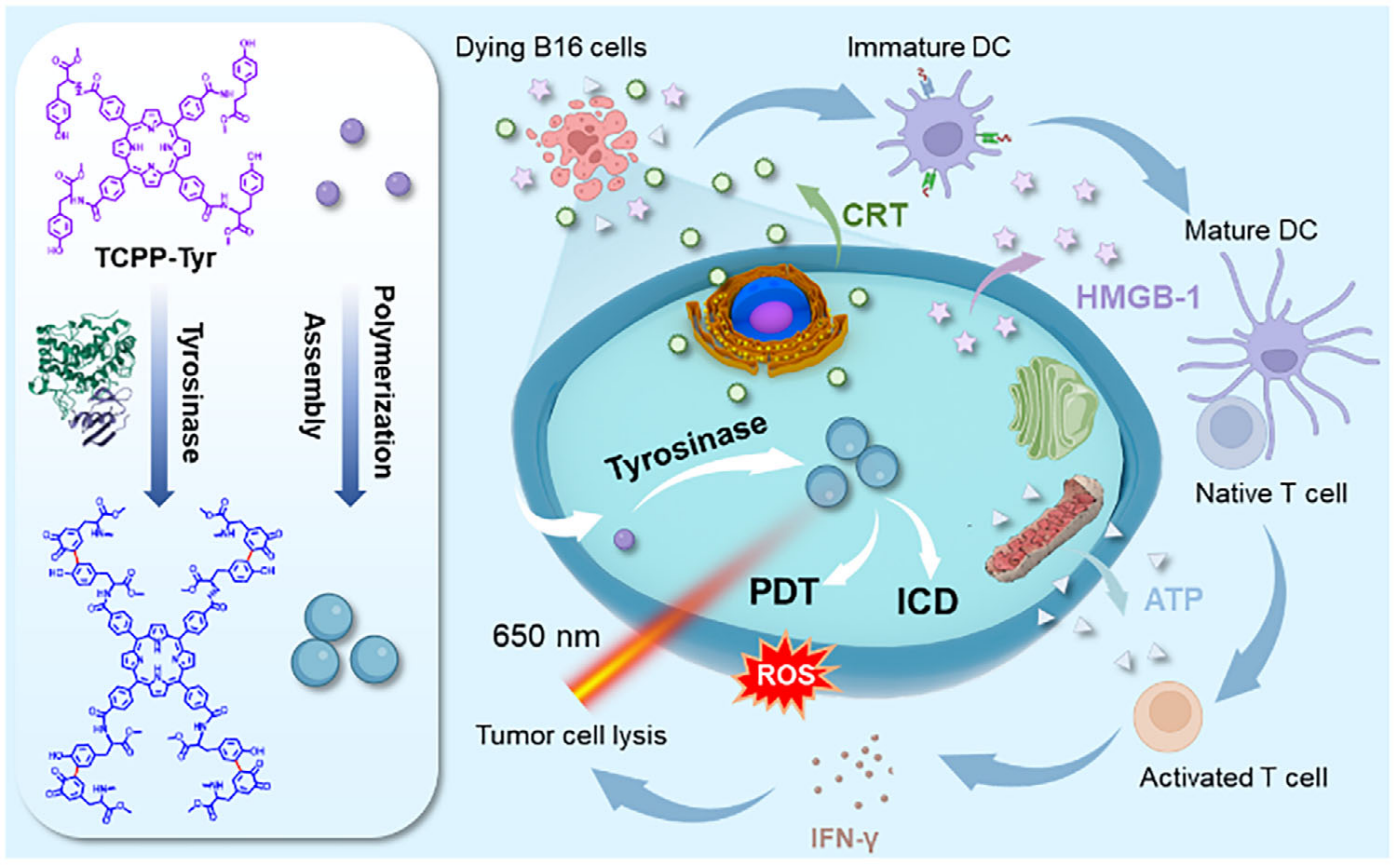
Schematic illustration of tyrosinase driven‐intracellular polymerization of a porphyrin derivative induced ICD of melanoma cells and strengthened PDT effect.

In this study, 5,10,15,20‐tetra (4‐methyl benzoyl‐L‐tyrosinate) porphyrin (TCPP‐Tyr) was designed and synthesized, in which four tyrosine molecules are conjugated to a single porphyrin. Using the overexpressed tyrosinase in melanoma cells, oxidative dimerization of tyrosine groups contributes to the intracellular biosynthesis of porphyrin‐based polymers. Furthermore, due to π‐π stacking and hydrophilic‐hydrophobic interactions, porphyrin‐based polymers assemble into micrometer‐scale aggregates, minimizing efflux of the porphyrin derivative from melanoma cells (Scheme 1). The intracellular formation of these aggregates induced immunogenic cell death (ICD) and promoted M1 macrophage polarization, dendritic cell maturation, and T cell activation. In addition, acting as an efficient photosensitizer [36, 37], the intracellular aggregates exhibited excellent photodynamic therapy (PDT) capability under near‐infrared light irradiation, further enhancing antitumor effects [38, 39, 40]. Assisted by anti‐PD‐L1 antibody (a‐PD‐L1) treatment, TCPP‐Tyr nearly eradicated tumors via intracellular polymerization, self‐assembly‐induced ICD, and strengthened PDT effects.

## 2 Results and Discussion

### 2.1 Tyrosinase‐Induced Polymerization of TCPP‐Tyr

**FIGURE 1:**
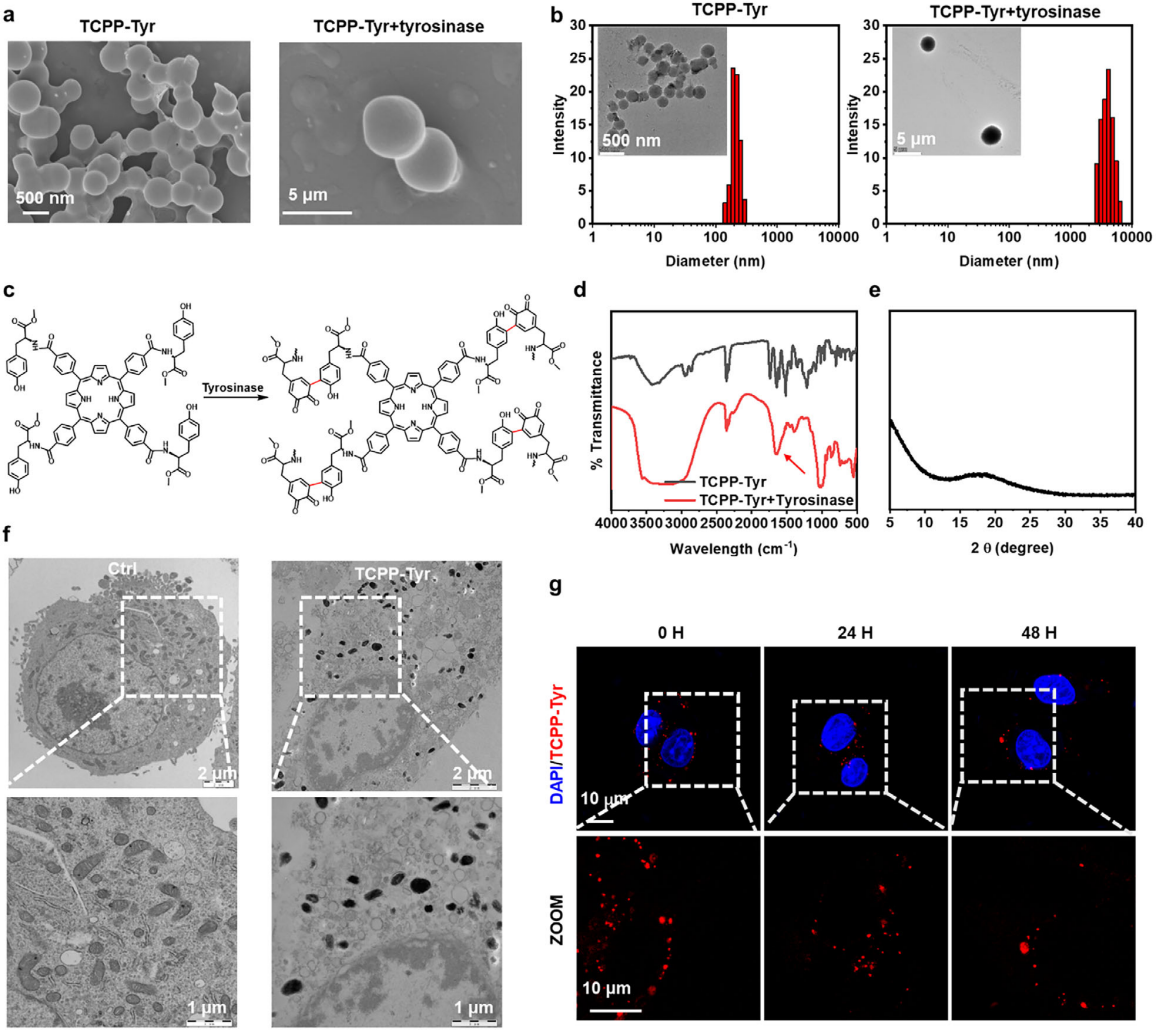
Tyrosinase‐driven polymerization and subsequent self‐assembly of TCPP‐Tyr. (a) SEM images of TCPP‐Tyr and TCPP‐Tyr+tyrosinase. (b) DLS experiments of TCPP‐Tyr and TCPP‐Tyr+tyrosinase. Inset: TEM images of TCPP‐Tyr and TCPP‐Tyr+tyrosinase. (c) Chemical structures of TCPP‐Tyr before and after oxidation. (d) FT‐IR spectra of TCPP‐Tyr and TCPP‐Tyr+tyrosinase. (e) XRD spectra of TCPP‐Tyr+tyrosinase. (f) Bio‐TEM of B16 cells incubated with PBS or TCPP‐Tyr. (g) CLSM images of B16 cells incubated with TCPP‐Tyr for 12 h, followed by culturing in fresh medium (without TCPP‐Tyr) for 0, 12, and 24 h.

To construct a fundamental building block for tyrosinase‐driven intracellular polymerization and PDT, a tyrosinefunctionalized porphyrin derivative, TCPP‐Tyr, was first synthesized through an amide condensation reaction between meso‐tetra(4‐carboxyphenyl)porphine and methyl L‐tyrosinate (Figure S1). The ^1^H NMR spectrum of TCPP‐Tyr exhibited a single peak at 3.735 ppm with an integral of 12 and a single peak at 9.275 ppm with an integral of 4, attributed to the methyl group of methyl L‐tyrosinate and the phenolic hydroxyl group in tyrosine methyl ester, respectively. These results demonstrated that four equivalents of methyl L‐tyrosinate were successfully grafted onto meso‐tetra (4‐carboxyphenyl) porphine (TCPP) (Figure S2), which was further confirmed by ^13^C NMR spectroscopy (Figure S3) and high‐resolution mass spectrometry (HRMS) (Figure S4). Subsequently, the morphology changes of TCPP‐Tyr undergoing dimerization upon tyrosinase oxidization were investigated by scanning electron microscopy (SEM). As shown in Figure 1a, TCPP‐Tyr formed spherical nanoparticles with an average diameter of ∼190 nm, likely resulting from hydrophilic and hydrophobic interactions and *π*–*π* accumulation. Meanwhile, the critical aggregation concentrations (CACs) of TCPP and TCPP‐Tyr (Figure S5) suggested that both compounds would assemble at extremely low concentrations (<1 µm). Following tyrosinase treatment, TCPP‐Tyr underwent oxidative polymerization via covalent coupling of oxidized tyrosine residues. The resulting polymer subsequently self‐assembled into micron‐sized aggregates with a diameter of ∼4.2 µm through non‐covalent interactions, including *π*–*π* stacking and hydrophobic effects. This size change of TCPP‐Tyr spheres with or without tyrosinase treatment was also observed using transmission electron microscopy (TEM) and dynamic light scattering (DLS) (Figure 1b). Under tyrosinase oxidation, tyrosine was transformed into the o‐quinone structure, which induced the dimerization reaction of the substituents proximal to the hydroxyl group on TCPP‐Tyr (Figure 1c). Fourier transform infrared spectroscopy (FT‐IR) showed a characteristic peak (1644.56 cm^−1^) corresponding to benzoquinone in TCPP‐Tyr after tyrosinase oxidation (Figure 1d; Figure S6), indicating successful dimerization. Furthermore, X‐ray diffraction (XRD) analysis showed a broad diffraction peak at 18° (Figure 1e), confirming the formation of polymer after tyrosinase oxidation.

In addition to high tyrosinase concentration, various cell types with or without tyrosinase overexpression were incubated with TCPP‐Tyr to assess its selective oxidation and polymerization. Biological transmission electron microscope (Bio‐TEM) images displayed distinct spherical nanoparticles, with an average diameter of ∼500 nm, within melanoma cells (B16 cells) after 12 h incubation with TCPP‐Tyr (Figure 1f). In contrast, human immortalized epidermal cells (HaCat cells), which do not overexpress tyrosinase, showed no formation of larger spherical particles (Figure S7). These findings indicated that the intracellular polymerization reaction of TCPP‐Tyr was highly selective in B16 cells that are known to have high concentrations of intracellular tyrosinase [41]. Upon incubation with TCPP‐Tyr for 12 h, confocal microscopy imaging showed that B16 cells exhibited strong red fluorescence from porphyrin, even after an additional 48 h. In contrast, B16 cells treated with TCPP lacking tyrosine units displayed negligible red fluorescence after only 24 h of incubation (Figure S8a). These observations were likely attributed to the rapid efflux of TCPP and the prolonged retention of polymerized TCPP‐Tyr aggregates in B16 cells due to tyrosinase‐drivenintracellular polymerization and self‐assembly (Figure 1g). Similarly, the red fluorescence in HaCat cells treated with TCPP‐Tyr was initially weaker than that in B16 cells and completely disappeared after 24 h (Figure S8b). This was attributed to the lack of adequate tyrosinase concentration to catalyze the oxidation reaction, leading to the rapid efflux of TCPP‐Tyr from HaCat cells. This experimental phenomenon was further verified by flow cytometry (Figure S9). The red fluorescence of TCPP‐Tyr could remain stable in B16 cells for up to 48 h, while it disappeared rapidly within 24 h in HaCat cells. The red fluorescence of TCPP gradually disappeared over time in B16 cells. Collectively, these results demonstrated that TCPP‐Tyr was able to undergo intracellular polymerization, due to catalysis by tyrosinase overexpressed in B16 cells, generating micron‐sized particles to prolong retention time in cells.

### 2.2 Intracellular Polymerization‐Induced Immunogenic Cell Death

**FIGURE 2:**
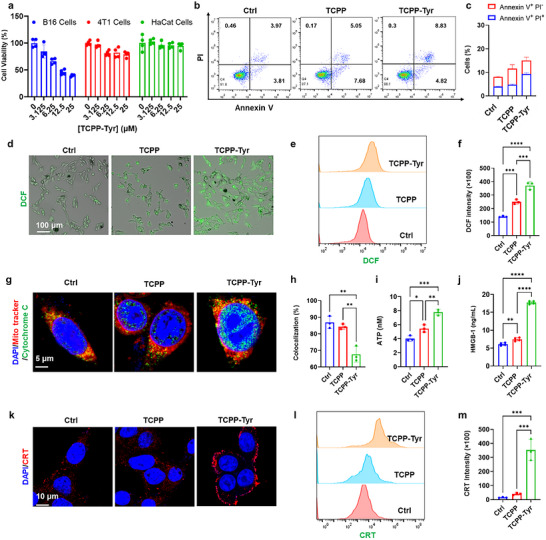
Intracellular polymerization of TCPP‐Tyr affected cellular behaviors and induced ICD of B16 cells. (a) Cell viability of B16 cells, 4T1 cells, and HaCat cells treated with different concentrations of TCPP‐Tyr for 12 h. The data were shown as mean value ± the standard deviation of independent experiments. One‐way ANOVA was utilized for statistical analysis. (b) Representative Annexin V‐FITC/PI co‐staining flow cytometry results and (c) the analytical results of B16 cells incubated with different treatments for 12 h. [TCPP‐Tyr] = [TCPP] = 6.25 µm. (d) Representative CLSM images of ROS generation in B16 cells as indicated by the green fluorescence of DCF that was oxidized from H2DCFDA by ROS. [TCPP‐Tyr] = [TCPP] = 6.25 µm. (e) Representative ROS staining flow cytometry results and (f) quantitative data of B16 cells with different treatments. [TCPP‐Tyr] = [TCPP] = 6.25 µm. The data were shown as mean value ± the standard deviation of independent experiments. Students’ t‐test was utilized for statistical analysis. Value of ^****^ *p* ≤0.0001 was applied to annotate statistical significance. (g,h) Release of cytochrome c from mitochondria in cells. Mitochondria (red fluorescence) and cytochrome c (green fluorescence) were stained by Mito. Tracker Red and anti‐cytochrome c antibody, respectively. The data were shown as mean value ± the standard deviation of independent experiments. Students’ t‐test was utilized for statistical analysis. Value of ^**^ *p* ≤0.01 was applied to annotate statistical significance. (i) Quantitative analysis of the ATP concentration in cell supernatants of B16 cells that received various treatments as indicated. [TCPP‐Tyr] = [TCPP] = 6.25 µm. The data were shown as mean value ± the standard deviation of independent experiments. Students’ t‐test was utilized for statistical analysis. Value of ^*^ *p* ≤0.05 was applied to annotate statistical significance. (j) Analysis of HMGB‐1 concentration in the supernatant of B16 cells after different treatments by ELISA. The data were shown as mean value ± the standard deviation of independent experiments. Students’ t‐test was utilized for statistical analysis. Value of ^**^ *p* ≤0.01 was applied to annotate statistical significance. (k) Representative CLSM images of CRT cell surface expression. (l) Representative CRT staining flow cytometry results and (m) quantitative data of B16 cells with different treatments. [TCPP‐Tyr] = [TCPP] = 6.25 µm. The data were shown as mean value ± the standard deviation of independent experiments. Students’ t‐test was utilized for statistical analysis. Value of ^**^ *p* ≤0.01 was applied to annotate statistical significance.

Given the selective intracellular polymerization and self‐assembly of TCPP‐Tyr in B16 cells, the effects on cell viability, function, and behavior were explored. B16 cells (tyrosinase activity: 4.03U/10^4^ cells), 4T1 cells, and HaCat cells were incubated with different concentrations of TCPP‐Tyr for 12 h [42]. Notably, B16 cell viability drastically decreased in a concentration‐dependent manner (Figure 2a). In contrast, TCPP‐Tyr caused negligible damage to the other two cell types. Furthermore, Annexin V and propidium iodide (PI) staining demonstrated that TCPP‐Tyr‐induced B16 cell death involved both apoptosis and necrosis (Figure 2b,c; Figure S10). Intracellular polymerization of TCPP‐Tyr increased the percentage of early apoptotic B16 cells (Annexin V^+^PI^−^) to 5.7% and late apoptotic/necrotic cells (Annexin V^+^PI^+^) to 9.3%, suggesting that intracellular polymerization of TCPP‐Tyr induced apoptosis and necrosis in B16 cells.

Reactive oxygen species (ROS) levels are essential for maintaining cellular homeostasis [43, 44]. Dichlorodihydrofluorescein diacetate (H_2_DCFDA) was utilized to detect ROS production via green fluorescence in B16 cells after intracellular polymerization. Confocal imaging showed prominent green fluorescence in B16 cells treated with TCPP‐Tyr (Figure 2d), which was further corroborated by quantitative flow cytometry analysis (Figure 2e,f). In contrast, HaCat cells showed little generation of ROS after incubation with TCPP‐Tyr (Figure S11), indicating that the intracellular polymerization of TCPP‐Tyr was responsible for ROS generation. The generation of large amounts of cellular ROS could trigger ICD [45, 46]. Accordingly, the relevant damage‐associated with molecular patterns (DAMPs) in TCPP‐Tyr‐treated B16 cells were investigated. Cytochrome c was released from mitochondria in B16 cells treated with TCPP‐Tyr, as evidenced by a significant decrease in colocalization between mitochondria (red) and cytochrome c (green) fluorescence (Figure 2g,h). Then, the culture supernatant of B16 cells treated with TCPP‐Tyr contained elevated concentrations of ATP (Figure 2i). The release of HMGB‐1 from B16 cells was quantified using enzyme‐linked immunosorbent assay (ELISA), revealing that TCPP‐Tyr treatment increased the release of HMGB‐1 (Figure 2j). Third, CRT protein predominantly localized in the cytoplasm of untreated B16 cells, while apparent translocation of CRT to the cell membrane was observed in B16 cells treated with TCPP‐Tyr (Figure 2k), as was further confirmed by quantitative flow cytometry (Figure 2l,m). Thus, the levels of DAMPs, such as CRT, extracellular ATP, and HMGB‐1, were all increased in B16 cells after TCPP‐Tyr treatment, indicating that intracellular polymerization of TCPP‐Tyr could induce ICD in B16 cells.

In addition, treatment with TCPP‐Tyr increased the percentage of B16 cells in G2/M phase arrest from 22.3% to 28.6% (Figure S12a), and intracellular polymerization specifically reduced the accumulation rate of S‐phase cells from 26.16% to 23.4% (Figure S12b). The migration ability of B16 cells treated with TCPP‐Tyr was greatly reduced in comparison to that of untreated B16 cells (Figure S13a), with nearly 40% of the wound area remaining unhealed even after incubation of 48 h (Figure S13b). Overall, the intracellular polymerization and assembly of TCPP‐Tyr selectively reduced the viability of B16 cells, induced DAMPs overexpression and ICD, and affected cell cycle as well as migration ability. These findings suggest a potential strategy for regulating tumor cell activity that differs from traditional drug‐based chemotherapy and immunotherapy.

### 2.3 Intracellular Polymerization Promoted Immune Cell Recognition and Activation

**FIGURE 3:**
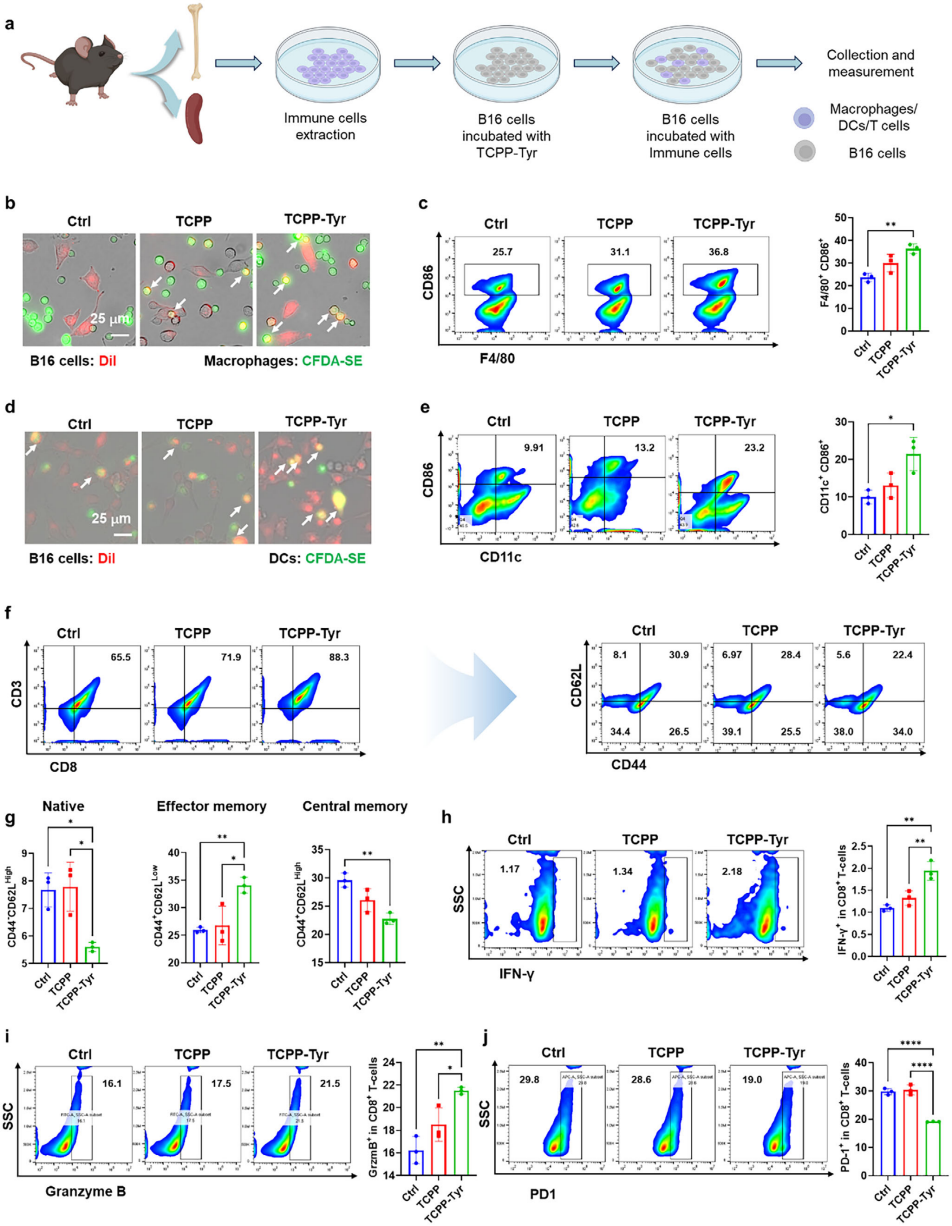
TCPP‐Tyr‐treated B16 cells facilitated M1 polarization of macrophages, DC maturation, and CD8^+^ T cell activation. (a) Schematic illustration of the co‐incubation process of immune cells with tumor cells. (b) CLSM images of macrophages incubated with B16 cells. Macrophages were stained with CFDA‐SE, green fluorescence. B16 cells were stained with Dil, red fluorescence. (c) Representative flow cytometry results and quantitative data of M1 phenotype (F4/80^+^ CD86^+^). The data were shown as mean value ± the standard deviation of independent experiments. Students’ t‐test was utilized for statistical analysis. Value of ^**^ *p* ≤0.01 was applied to annotate statistical significance. (d) CLSM images of DCs incubated with B16 cells. DCs were stained with CFDA‐SE, green fluorescence. B16 cells were stained with Dil, red fluorescence. (e) Representative flow cytometry results and quantitative data of mature DCs (CD11c^+^ CD86^+^). The data were shown as mean value ± the standard deviation of independent experiments. Students’ t‐test was utilized for statistical analysis. Value of ^*^ *p* ≤0.05 was applied to annotate statistical significance. (f) Representative flow cytometry results of T cells in co‐incubated cells. (g) Quantitative data of CD8^+^ T cells with naive (CD44^−^CD62L^high^), central memory (CD44^+^CD62L^high^), and effector memory (CD44^+^CD62L^low^) phenotypes. The data were shown as mean value ± the standard deviation of independent experiments. Students’ t‐test was utilized for statistical analysis. Value of ^**^ *p* ≤0.01 and ^***^ *p* ≤0.001 were applied to annotate statistical significance. (h) Representative flow cytometry results and quantitative data of the content of IFN‐γ secreted by CD8^+^ T cells. The data were shown as mean value ± the standard deviation of independent experiments. Students’ t‐test was utilized for statistical analysis. Value of ^*^ *p* ≤0.05 was applied to annotate statistical significance. (i) Representative flow cytometry results and quantitative data of the content of Granzyme B secreted by CD8^+^ T cells. The data were shown as mean value ± the standard deviation of independent experiments. Students’ t‐test was utilized for statistical analysis. Value of ^**^ *p* ≤0.01 was applied to annotate statistical significance. (j) Representative flow cytometry results and quantitative data of the content of PD‐1 content expressed on CD8^+^ T cells surfaces. The data were shown as mean value ± the standard deviation of independent experiments. Students’ t‐test was utilized for statistical analysis. Value of ^****^ *p* ≤0.0001 was applied to annotate statistical significance.

After confirming that intracellularly synthesized polymers could induce ICD, the interactions between TCPP‐Tyr‐treated B16 cells and immune cells were investigated. As shown in Figure 3a, macrophages and dendritic cells (DCs) were isolated from the bone marrow, while CD8^+^ T cells were isolated from the spleens of 6‐week‐old female C57BL/6 mice. To assess the immunogenicity of B16 cells after intracellular polymerization, the internalization and activation of macrophages and DCs in response to B16 cells treated with TCPP‐Tyr, the activation of T cells, and the release of lymphocyte‐associated cytokines were analyzed. B16 cells were incubated with TCPP‐Tyr for 12 h and subsequently labeled with Dil (red fluorescence). An equal number of macrophages labeled with CFDA‐SE (green fluorescence) were co‐cultured with the treated B16 cells for 6 h. As shown in Figure 3b, macrophages co‐cultured with TCPP‐Tyr‐treated B16 cells exhibited large areas of overlapping red and green fluorescence in comparison to those incubated with untreated B16 cells, indicating intracellular polymerization greatly promoted the phagocytosis of B16 cells by macrophages. Furthermore, macrophages co‐incubated with TCPP‐Tyr‐treated B16 cells exhibited significantly increased CD86 expression, contributing to M1 polarization (Figure 3c). Similarly, B16 cells treated with TCPP‐Tyr were readily recognized and internalized by DCs (Figure 3d), and co‐inoculation contributed to a significant increase in CD86 expression of DCs, indicating a higher maturation rate of DCs (Figure 3e). Finally, tumor cells were incubated with T cells at a 1:10 ratio for 12 h, followed by CD8^+^ T cells being selected from the mixed cells and analyzed by flow cytometry (Figure 3f). As shown in Figure 3f,g, co‐incubation with B16 cells treated with TCPP‐Tyr resulted in a 1.4‑fold increase in the proportion of effector memory T cells (CD44^+^CD62L^low^CD8^+^ T cells) increased significantly by 1.4 times, compared to co‐incubation with untreated B16 cells. It was also observed that the levels of IFNγ and Granzyme B produced by CD8^+^ T cells co‐cultured with treated B16 cells increased by 1.2‐fold and 1.4‐fold, respectively, compared to those co‐cultured with untreated B16 cells (Figure 3h,i). CD8^+^ T cells co‐inoculated with TCPP‐Tyr‐treated B16 cells exhibited a 40% down‐regulation in programmed death receptor 1 (PD‐1) expression, compared to those co‐incubated with untreated B16 cells, indicating that TCPP‐Tyr treatment improved the release of lymphocyte‐associated cytokines in CD8^+^ T cells (Figure 3j). Collectively, intracellular polymerization of TCPP‐Tyr induced ICD and enhanced the immunogenicity of B16 cells, facilitating to their internalization by macrophages and DC, M1 polarization of macrophage, DC maturation, and CD8^+^ T cell activation.

### 2.4 Selective Intracellular Polymerization and Retention of TCPP‐Tyr in Tumors

**FIGURE 4:**
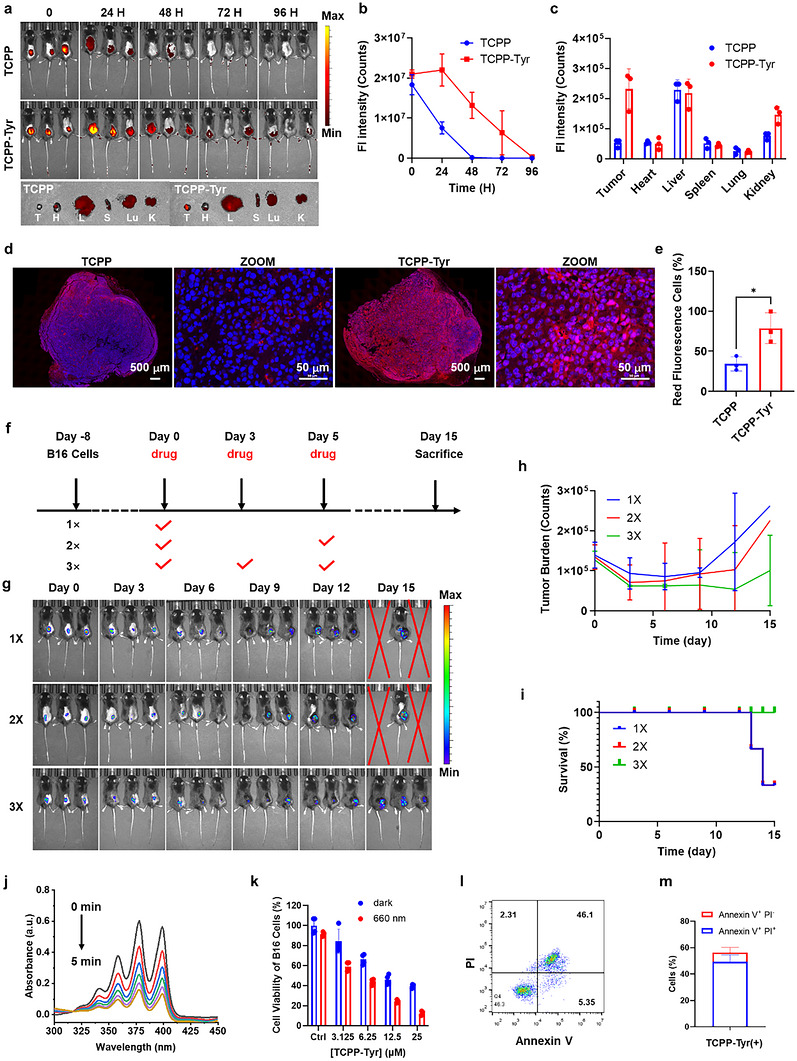
TCPP‐Tyr selectively polymerized in melanoma tissue and exhibited a tumor inhibition effect. (a) IVIS bioluminescent images of B16 tumor‐burden mice injected with TCPP or TCPP‐Tyr, [TCPP] = [TCPP‐Tyr] = 6.25 µm, 100 µL for different durations (0, 24, 48, 72, and 96 h). After administration for 96 h, ex vivo fluorescence imaging was conducted on heart (H), liver (L), spleen (S), lungs (Lu), kidneys (K), and tumors (T) from treated mice. (b) The changes of fluorescence intensity (FI) in the tumor after administration for 96 h. (c) The changes of relative fluorescence intensity (FI) in heart, liver, spleen, lungs, kidneys, and tumors from the treated groups. (d) Fluorescence imaging of tumors in the treated groups. DAPI (blue), TCPP (red), or TCPP‐Tyr (red). (e) Semi‐quantitative analysis of the red fluorescent cells. The data were shown as mean value ± the standard deviation of independent experiments. Students’ t‐test was utilized for statistical analysis. Value of ^*^ *p* ≤0.05 was applied to annotate statistical significance. (f) Schematic illustration of an antitumor experiment in mice. (g) IVIS bioluminescent images of B16 tumor growth for 15 days (received 1 – 3 injections of TCPP‐Tyr, [TCPP‐Tyr] = 25.0 µm, 100 µL). (h) Graph of tumor burden (Counts) across time. (i) Survival rate curve of the mice treated different times for 15 days. (j) UV–vis spectra of ABDA in the presence of TCPP‐Tyr under 650 nm light irradiation. The decreased UV–vis absorbance indicates that ABDA was gradually consumed by the photochemically generated ^1^O_2_. ([TCPP‐Tyr] = 25.0 µm, [ABDA] = 50.0 µm, 2.5% DMSO in PBS). (k) Cell viabilities of B16 cells treated with TCPP‐Tyr with or without light irradiation. (l) Representative Annexin V‐FITC/PI co‐staining flow cytometry results and (m) the analytical results of B16 cells incubated with TCPP‐Tyr and light irradiation. [TCPP‐Tyr] = 6.25 µm.

Given that intracellular polymerization of TCPP‐Tyr induced cell death and improved the immunogenicity of tumor cells, the efficacy of this strategy in antitumor treatment was further investigated. First, melanoma‐bearing mice were intratumorally administered with TCPP‐Tyr or the control molecule TCPP. The change of fluorescence intensity was detected by in vivo imaging system (IVIS). As shown in Figure 4a,b, the fluorescence signal of TCPP group diminished significantly 48 h after administration, while the red fluorescence at the tumor site in TCPP‐Tyr‐treated mice persisted for up to 96 h, indicating that intracellular polymerization of TCPP‐Tyr formed large‐sized particles and extended its retention time in tumor tissue. Furthermore, another batch of treated mice were euthanized 48 h post‐administration, and the biodistribution in organs and tumors was further assessed through ex vivo fluorescence imaging. Figure 4a,c showed that red fluorescence was mainly concentrated at the tumor site in TCPP‐Tyr‐treated mice, while fluorescence appeared in the liver and kidneys was a normal physiological phenomenon in the metabolic process. Subsequently, tumor sections were prepared and fluorescently imaged at the cellular level (Figure 4d,e). TCPP‐Tyr treated mice exhibited 74% red fluorescence coverage in the entire tumor section, while only 31.9% coverage was observed in TCPP‐treated mice, demonstrating the selective intracellular polymerization and retention of TCPP‐Tyr in the melanoma tumor. Magnified fluorescence images further confirmed the efficient polymerization of TCPP‐Tyr within B16 cells (Figure 4d).

### 2.5 Intracellular Polymerization of TCPP‐Tyr Inhibited Melanoma Growth

After confirming the efficient polymerization, prolonged retention, and induction of ICD by TCPP‐Tyr in melanoma, the antitumor therapeutic effect of TCPP‐Tyr was investigated. Mice were subcutaneously inoculated with B16 cells and, after eight days, received 1–3 intratumoral injections of TCPP‐Tyr. During this period, tumor growth conditions were monitored using IVIS (Figure 4f). While this system could be potentially administered intravenously, the relatively low drug concentration at the tumor site may limit its therapeutic efficacy [6]. As shown in Figure 4g–i, the tumor growth was partially inhibited after one or two doses of drug treatment, but prolonged observation revealed a risk of tumor recurrence leading to mortality. However, the administration of three doses of TCPP‐Tyr largely inhibited the growth of mouse tumors, and greatly improved survival rates. These results confirmed that TCPP‐Tyr exhibited antitumor activity against melanoma due to selective intracellular polymerization.

### 2.6 Intracellular Polymerization of TCPP‐Tyr Enhanced Photodynamic Therapy

Porphyrin is well known as an efficient photosensitizer for PDT, thus its combination with intracellular polymerization was expected to enhance tumor suppression. 9,10‐Anthracenediyl‐bis(methylene)‐dimalonic acid (ABDA) was utilized as an indicator to evaluate the ROS generation ability of TCPP‐Tyr under 650 nm irradiation (1.0 W/cm^2^). The absorption peak of ABDA at 378 nm gradually decreased with prolonged irradiation, and the corresponding degradation curve of ABDA (Figure 4j; Figure S14) demonstrated efficient ROS generation, highlighting the potential of TCPP‐Tyr for PDT applications. The ability of TCPP‐Tyr to produce singlet oxygen under red light irradiation after undergoing oxidative polymerization was not significantly changed. Then, a quantitative assessment of the PDT effect on B16 cells was conducted. After incubation with TCPP‐Tyr for 12 h, only B16 cells showed a concentration‐dependent decrease in cell viability in comparison to 4T1 and HaCat cells (Figure S15), attributed to tyrosinase‐driven intracellular polymerization. Upon 5 min of 650 nm irradiation, the viability of B16 cells was further reduced by more than 20% (Figure 4k), suggesting the photodynamic properties of the intracellular porphyrin‐based polymer contributed to further inhibition of B16 cell growth. Flow cytometry analysis exhibited an increase in late apoptotic/necrotic cells (Annexin V^+^PI^+^) from 9.3% to 49.2%, compared to B16 cells without irradiation (Figures 2b and 4l,m; Figure S15). However, the cytotoxicity caused by light on TCPP‐Tyr‐treated 4T1 and HaCat cells was essentially the same, which is due to the photophysical properties of TCPP‐Tyr itself (Figure S16). As shown in Figure S17a, B16 cells treated with TCPP and TCPP‐Tyr produced a large amount of ROS after further light exposure. Quantitative results from flow cytometry indicated that the two treatment methods did not result in a significant difference in the ROS levels produced in the cells. This might be due to the higher negative charge density on the TCPP surface, making it less readily internalized than TCPP‐Tyr (Figure S17b). Therefore, intracellular polymerization of TCPP‐Tyr inhibited the efflux of porphyrin derivative, offering a combined therapeutic approach of PDT and intracellular polymerization‐mediated ICD to inhibit tumor cell growth.

### 2.7 TCPP‐Tyr Combined with PDT and a‐PD‐L1 Strengthened Antitumor Efficacy

**FIGURE 5:**
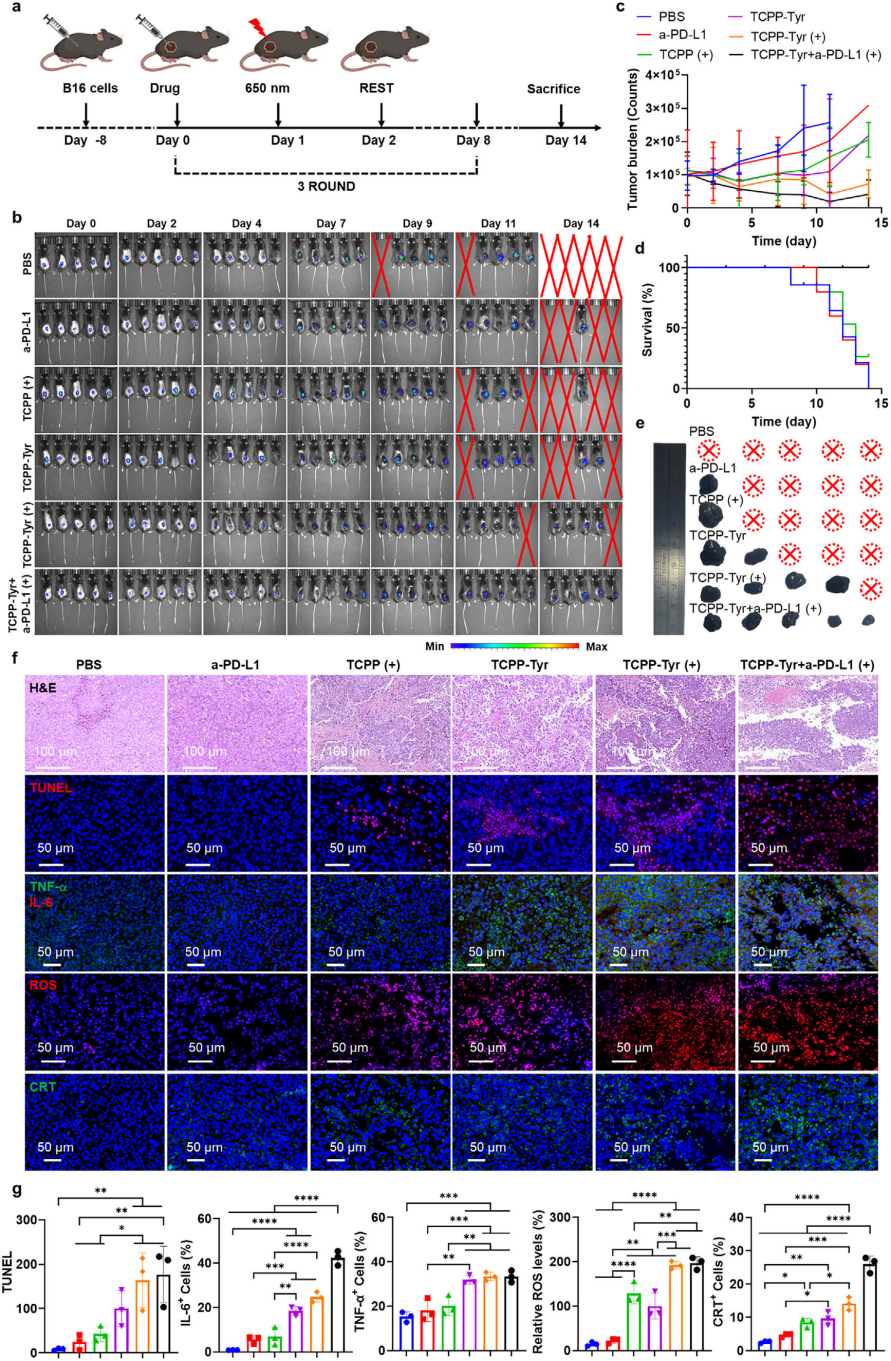
TCPP‐Tyr+a‐PD‐L1 (+) effectively inhibited tumor growth accompanied by ICD. (a) Schematic illustration of an antitumor experiment in mice. (b) IVIS bioluminescent images of B16 tumor growth for 15 days. (c) Graph of tumor burden (Counts) across time of control and treated mice. (d) Survival rate curve of the mice treated different times. (e) Representative photos of the excised tumors on day 14 after the various treatments. A circle with a cross indicated that the tumor volume of the mouse reached the threshold for euthanasia or that the mouse died naturally during treatment. (f) The representative micrographs of H&E, TUNEL, TNF‐α&IL‐6, ROS, and CRT tracker‐stained tumor slices 24 h after the third corresponding treatment. (g) Semi‐quantitative analysis of TUNEL, IL‐6, TNF‐α, ROS, and CRT of the tumor slices. The data were shown as mean value ± the standard deviation of independent experiments. One‐way ANOVA was utilized for statistical analysis. Values of ^*^ *p* ≤0.05, ^**^ *p* ≤0.01 ^***^ *p* ≤0.001, and ^****^ *p* ≤0.0001 were applied to annotate statistical significance.

TCPP‐Tyr exhibited potential antitumor effects via PDT and immunotherapy through intracellular polymerization. To improve the immunotherapeutic effect, the immune adjuvant (a‐PD‐L1) was added. Tumor‐bearing mice were randomly divided into 6 groups: PBS, a‐PD‐L1, TCPP with laser (TCPP (+)), TCPP‐Tyr, TCPP‐Tyr with laser (TCPP‐Tyr (+)), and TCPP‐Tyr+a‐PD‐L1 with laser (TCPP‐Tyr+a‐PD‐L1 (+)) with a total of three doses (Figure 5a). PBS, TCPP, and TCPP‐Tyr were injected intratumorally, and a‐PD‐L1 was injected intraperitoneally. After treatment with TCPP or TCPP‐Tyr for 24 h, the tumor was irradiated with 650 nm (1.0 W/cm^2^) laser for 5 min. As shown in Figure 5b–e and Figure S18, tumors in PBS group grew rapidly, and all mice died by the end of the experiment (15 days). A‐PD‐L1 treatment also demonstrated a negligible therapeutic effect, comparable to the PBS group, indicating that the immune checkpoint inhibitor alone could not effectively inhibit tumor growth. Mice treated with TCPP (+) showed very modest tumor growth inhibition, likely due to the cellular efflux of TCPP resulting in a weaker PDT effect. In contrast, TCPP‐Tyr treatment resulted in a 40% survival rate, with significant tumor growth inhibition, and the overall survival rate was further increased to 80% with laser irradiation. With the addition of a‐PD‐L1, TCPP‐Tyr+a‐PD‐L1 (+) showed the best antitumor performance, effectively inhibiting tumor growth and achieving 100% survival during the observation period (Figure 5d). These findings suggested that TCPP‐Tyr induced ICD and strengthened PDT due to intracellular polymerization, and when combined with immune checkpoint inhibition, significantly inhibited the growth of tumors and improved the survival rate of mice bearing melanoma.

Histological hematoxylin and eosin (H&E) staining, TUNEL staining, and TNF‐α&IL‐6 staining were performed on tumor tissue sections to evaluate tumor cell apoptosis, necrosis, and inflammation. As shown in Figure 5f,g, the most severe tumor cell death and the most severe inflammation could be clearly observed in the TCPP‐Tyr+a‐PD‐L1 (+) group. Then, the intratumoral ROS generation in different treatment groups was detected with DCFH‐DA probe. The TCPP‐Tyr+a‐PD‐L1 (+) group showed intense green fluorescence in comparison to other groups, indicating a high ROS generation. To evaluate whether it was effective in eliciting an immune response, CRT was examined as an important marker of ICD. Fluorescence microscopy images and semi‐quantitative analysis displayed no apparent signals for CRT in the PBS and TCPP (+) groups. Moderate CRT translocation was detected in the TCPP‐Tyr and TCPP‐Tyr (+) groups, whereas a marked enhancement in CRT signals was observed in the TCPP‐Tyr+a‐PD‐L1 (+) group. Therefore, intracellular polymerization of TCPP‐Tyr combined with PDT induced severe tumor damage and enhanced tumor immunogenicity, potentially eliciting an antitumor immune response.

### 2.8 TCPP‐Tyr+a‐PD‐L1 (+) Treatment Activated Strong Immune Response

**FIGURE 6:**
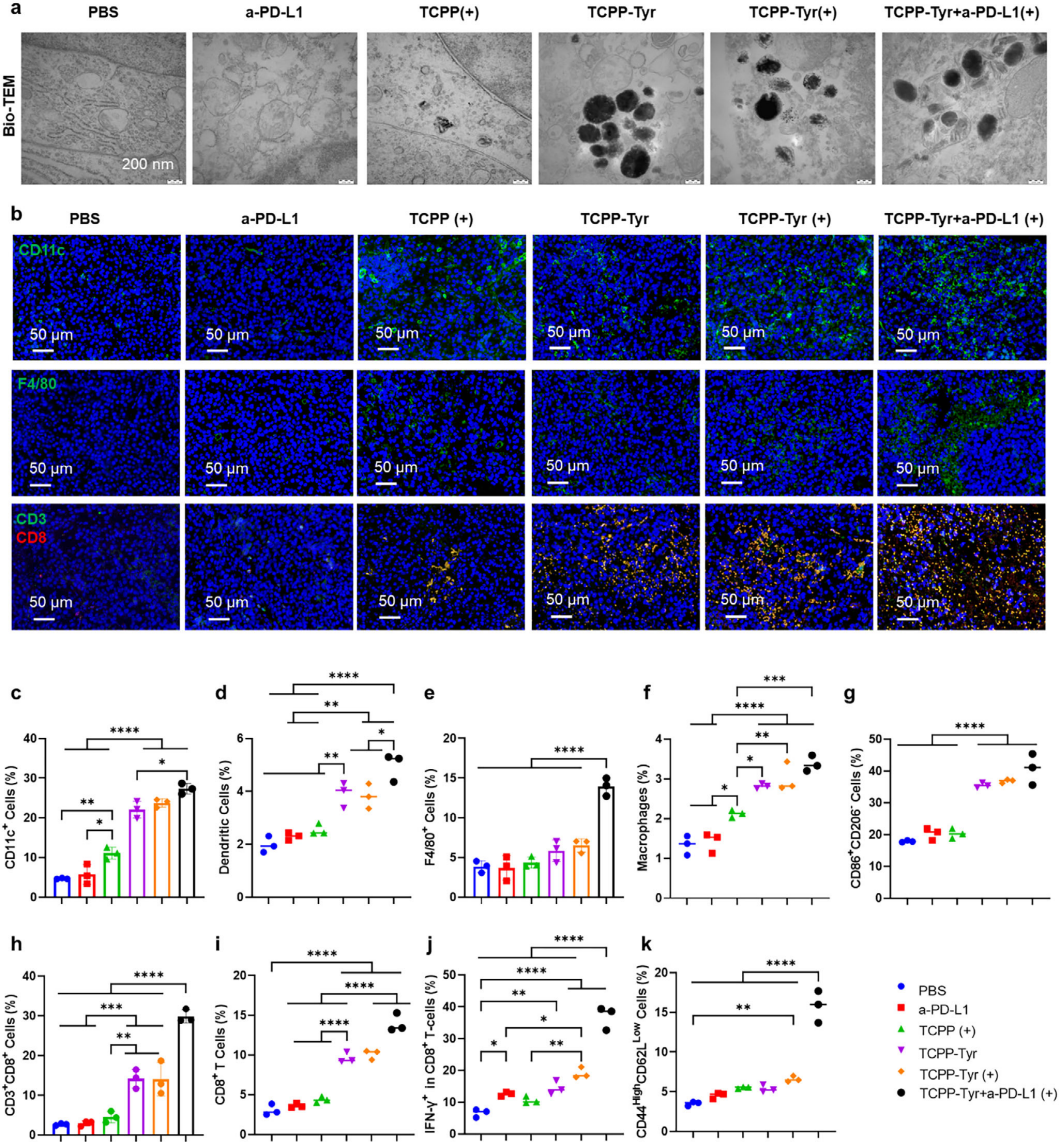
TCPP‐Tyr+a‐PD‐L1 (+) formed intracellular micron‐sized particles and contributed to a strong immune activation micro‐environment in melanoma‐bearing mice. (a) The representative Bio‐TEM images of tumor tissues 24 h after the third corresponding treatment. (b) Immunofluorescence analysis showing the infiltration of DCs, macrophages, and T cells 24 h after the third corresponding treatment. The densities of (c) CD11c^+^, (e) F4/80^+^, and (h) CD3^+^CD8^+^cells in the whole tumors 24 h after the third corresponding treatment, from the confocal images of immunofluorescence staining (n = 3). The percentages of (d) DCs, (f) total macrophages, (g) M1 type macrophages, (i) CD8^+^ T cells, (j) IFN‐γ^+^ cells, and (k) CD44^high^CD62L^low^ cells by flow cytometry 24 h after the third corresponding treatment. The data were shown as mean value ± the standard deviation of independent experiments. One‐way ANOVA was utilized for statistical analysis. Values of ^*^ *p* ≤0.05, ^**^ *p* ≤0.01 ^***^ *p* ≤0.001, and ^****^ *p* ≤0.0001 were applied to annotate statistical significance.

Since intracellular polymerization of TCPP‐Tyr was confirmed to kill tumor cells and induce ICD, the in vivo intracellular polymerization process and immune activation were further investigated. Bio‐TEM imaging on tumor section from treated mice showed that large spherical particles were formed in mice treated with TCPP‐Tyr, TCPP‐Tyr (+), and TCPP‐Tyr+a‐PD‐L1 (+), indicating in vivo intracellular polymerization of TCPP‐Tyr (Figure 6a; Figure S19). Immuno‐fluorescence staining was used to assess tumor immune cell infiltration (Figure 6b), and the first step in the immune response process was recognizing and processing of tumor antigens by DCs. On the third day after treatment, it could be clearly seen that the tumor infiltration of DCs (CD11c^+^ cells) increased in mice treated with TCPP‐Tyr (Figure 6b,c), as confirmed by flow cytometric analysis (Figure 6d; Figure S20). In addition to DCs, macrophages (F4/80^+^ cells) also showed high tumor infiltration, as determined by immunofluorescence staining (Figure 6b,e) and flow cytometry (Figure 6f; Figure S21). These cells were able to engulf and digest their own senescent and abnormal cells, and secret pro‐inflammatory cytokines to recruit other immune cells. As expected, TCPP‐Tyr+a‐PD‐L1 (+) treatment induced the highest infiltration rate of DCs and macrophages in tumor tissues. The enhanced infiltration of DCs and macrophages in tumors indicated that intracellular polymerization effectively improved immunogenicity and achieved effective antigen presentation. In addition, macrophages in mice treated with TCPP‐Tyr+a‐PD‐L1 (+) were significantly polarized toward the M1 type (Figure 6g; Figure S21).

When tumor antigens were presented to T cells, they were activated to produce a specific immune response for anti‐tumor immunotherapy. Immunofluorescence staining showed a significant increase in the proportion of infiltrating CD3^+^CD8^+^ cells in mice treated with TCPP‐Tyr (Figure 6b,h), demonstrating an increased proportion of cytotoxic T cells in the tumor microenvironment. Flow cytometry analysis confirmed an increased number of CD8^+^ T cells in the tumors of mice treated with TCPP‐Tyr, with further infiltration of CD8^+^ T cells resulting from combination with light irradiation and a‐PD‐L1 (Figure 6i; Figure S22). Meanwhile, the proportion of lymphocyte factor (antigen‐specific IFN‐γ) was significantly increased in mice treated with TCPP‐Tyr (Figure 6j; Figure S22), and TCPP‐Tyr+a‐PD‐L1 (+) treatment induced the highest expression of IFN‐γ in CD8^+^ T cells. These results indicated that intracellular polymerization effectively induced tumor‐specific T cell responses, which were further enhanced by the combination treatment of a‐PD‐L1 and light irradiation. Moreover, the proportion of effector memory T cells (CD44^high^CD62L^low^) increased significantly in the tumors of mice treated with TCPP‐Tyr+a‐PD‐L1 (+) (Figure 6k; Figure S22), indicating that intracellular polymerization of TCPP‐Tyr, in combination with PDT and an immune checkpoint inhibitor, had the potential to induce long‐term memory responses with durable anti‐tumor effects.

A bilateral subcutaneous melanoma model in C57BL/6 mice was established, followed by treatment of only the right‐sided tumor, to evaluate the systemic immune activation effects due to intracellular polymerization and photodynamic therapy (Figure S23a). During the observation period, there was no significant decrease in body weight in any treatment group (Figure S23b), and the TCPP‐Tyr+a‐PD‐L1 (+) treatment group significantly improved the survival rate of mice (Figure S23c). Furthermore, TCPP‐Tyr+a‐PD‐L1 (+) treatment significantly slowed the proliferation of the left (untreated) tumor, compared to the treatment group with TCPP+a‐PD‐L1 (+), which was unable to undergo intracellular aggregation (Figure S23d,e).

To evaluate the histopathological toxicity associated with intracellular polymerization, H&E staining analysis was performed on the heart, liver, spleen, lungs, and kidneys of all treated mice. As shown in Figure S24, no significant physiological morphological changes were observed in any of the organs, indicating that the selective intracellular polymerization of TCPP‐Tyr in melanoma tissue exhibited favorable biological safety. Furthermore, whole blood and serum were collected from mice treated with PBS and TCPP‐Tyr+a‐PD‐L1 (+) to conduct the blood routine analysis and assessment of related liver and kidney function. There was no significant alteration in blood routine (WBC, RBC, HGB, HCT, MCV, MCH, RDW, MPV, PLT, Lym# and Lym%), and liver and kidney functions (ALT, AST, TP, CREA, and BUN) after TCPP‐Tyr+a‐PD‐L1 (+) treatment (Figure S25). These findings showed that the selective intracellular polymerization of TCPP‐Tyr exhibited a high safety profile, which only induced a strong antitumor immune response in melanoma‐bearing mice and achieved favorable therapeutic effects when combined with PDT and immune checkpoint inhibition.

## 3 Conclusions

In summary, this study developed a tumor treatment strategy involving in situ intracellular polymerization and self‐assembly of a newly designed porphyrin derivative, via inducing ICD in B16 cells and strengthening PDT. Based on the biosynthesis of melanin, a tyrosine‐modified porphyrin (TCPP‐Tyr) was designed. In B16 cells, overexpressed tyrosinase catalyzed the oxidative polymerization of TCPP‐Tyr, followed by supramolecular self‐assembly to form micrometer‐sized spherical nanoparticles, which effectively inhibited its efflux and improved its intracellular retention. The intracellular aggregates of TCPP‐Tyr polymers in B16 cells led to ROS generation, apoptosis, necrosis, and migration inhibition, and induced the hallmark signals of ICD, including ATP release and HMGB‐1 overexpression, cytochrome c leaked, CRT inverted on the cell membrane, and cell cycle arrest. Accordingly, TCPP‐Tyr treated B16 cells effectively promoted the M1 polarization of macrophage, DC maturation, and the T cell activation, thereby contributing to the antitumor immunotherapy. In vivo experiments demonstrated that TCPP‐Tyr selectively polymerized and self‐assembled in the melanoma tumor, thereby exerting prolonged retention and tumor growth inhibition. Given that porphyrin is an excellent photosensitizer, PDT was further induced by laser irradiation to enhance the anti‐tumor immunotherapy, resulting in a remarkable improvement in the survival rate of melanoma‐bearing mice. When combined with a‐PD‐L1, TCPP‐Tyr (+) exhibited the best performance in eliminating tumors, accompanied by tumor cell ICD, immune cell infiltration, M1 macrophage polarization, DC maturation, CD8^+^ T cell activation, immune checkpoint inhibition, and ROS generation.

The key innovation of this manuscript lies in utilizing an endogenous enzyme (tyrosinase) within tumor cells to drive the specific, in situ intracellular polymerization and self‐assembly of photosensitizers. Most existing supramolecular nanoparticle strategies involve pre‐synthesizing nanoparticles extracellularly with specific structures and delivering them to the tumor site via blood circulation or local injection, relying on the EPR effect or active targeting to enter cells. [4, 15, 19]. In contrast, this work delivers a small monomer (TCPP‐Tyr) with a low critical aggregation concentration (Figure S6), forming temporary assemblies in body fluids to aid delivery. Subsequently, the key functional polymer is generated in situ within the target cells, triggered by specific endogenous enzymes (tyrosinases). Furthermore, existing nanoparticles, whether passively or actively targeted, still face the risk of efflux or lysosomal degradation after entering the cancer cells, resulting in limited retention time. In this strategy, the tyrosinase‐catalyzed oxidative polymerization reaction is irreversible. The resulting polymer further self‐assembles into micron‐sized aggregates through *π*–*π* stacking and hydrophobic interactions, making them resistant to cellular clearance, leading to long‐term, precise retention within the target cells (Figures 1g and 4a–c). From the perspective of targeting specificity, the specificity of traditional actively targeted nanoparticles depends on the overexpression of receptors on the cell surface [47, 48, 49]. However, many tumor‐associated receptors are also expressed to some extent on normal cells, potentially leading to off‐target effects. In contrast, the specificity of this work relies on the overexpression and catalytic activity of intracellular functional enzymes (tyrosinases), with this targeting occurring within the cell. Only melanoma cells with high tyrosinase activity can effectively convert TCPP‐Tyr monomers into polymer aggregates. For normal cells with low enzyme activity (such as HaCat), monomers cannot polymerize efficiently and are thus rapidly expelled from the cells (Figure S8b). Collectively, this design leads to high selectivity and safety (Figures S22 and S23).

Although the therapeutic efficacy of in situ intracellular polymerization is promising, comprehensive and in‐depth studies are essential, especially for the assessment of pharmacokinetics and stability. This is important for understanding the circulation and metabolic pathways of TCPP‐Tyr and supporting clinical technology transformation. Moreover, although this strategy exhibited excellent tumor cell selectivity without causing obvious damage to healthy tissues, the lack of a tumor‐targeting group precludes its use via intravenous injections, a limitation that still needs further exploration and optimization in the future. Meanwhile, while our study focuses on the “output” (intratumoral invasion), the “input” (draining lymph node initiation) remains a critical component for future mechanistic investigation. In the future, single‐cell RNA sequencing research will further refine our findings. Nonetheless, the findings of this study provide new insights into antitumor therapy through endogenous enzyme‐driven intracellular polymerization, offering a valuable alternative to current drug delivery and targeted therapy.

## Conflicts of Interest

The authors declare no conflicts of interest.

## Supporting information




**Supporting File**: advs75353‐sup‐0001‐SuppMat.docx.

## Data Availability

The data that support the findings of this study are available in the Supporting Information of this article.
