# Supplementary material for: Tyrosinase Driven‐Intracellular Polymerization of a Porphyrin Derivative Induced Immunogenic Death of Melanoma Cells and Strengthened Photodynamic Therapy
Source: Adv Sci (Weinh). 2026 Apr 20;13(39):e75353. doi: 10.1002/advs.75353 (PMC13335602; doi:10.1002/advs.75353)
Supplement: Supplementary file 1 — Supporting File: advs75353‐sup‐0001‐SuppMat.docx. [file ADVS-13-e75353-s001.docx]

**Supporting information**

**Tyrosinase driven-intracellular polymerization of a porphyrin derivative induced immunogenic death of melanoma cells and strengthened photodynamic therapy**

Mian Tang^1^, Junteng Qiu^1^, Yunfeng Lu^1^, Yahui Song^1^, Xinyu Zhou^1^, Tianshun Duan^1^, Xuanqi Peng^1^, Caixia Yin,^2*^ Cheng Gao^1,3*^, and Ruibing Wang^1*^

^1^ State Key Laboratory of Mechanism and Quality of Chinese Medicine, Institute of Chinese Medical Sciences, and MoE Frontiers Science Center for Precision Oncology, University of Macau, Taipa, Macau SAR 999078, China

^2^ Key Laboratory of Chemical Biology and Molecular Engineering of Ministry of Education, Institute of Molecular Science, Shanxi University, Taiyuan 030006, China

^3^ School of Pharmacy, Shenzhen University Medical School, Shenzhen University, Shenzhen, 518055, PR China

E-mail: yincx@sxu.edu.cn; [chenggao@szu.edu.cn](mailto:chenggao@szu.edu.cn); rwang@um.edu.mo

**Experimental section**

**Materials.** All chemicals were commercially available reagent grade and used without further purification, unless otherwise noted.

**Instruments.** NMR spectra were recorded on Bruker 600 MHz instrument, and chemical shifts were recorded in parts per million (ppm). TEM images were acquired by FEI Tecnai F20, America. The samples were prepared by placing a drop of solution onto a carbon-coated copper grid and air-dried. Transmission spectra and UV-vis absorption spectra were recorded on UV–vis spectrophotometer (DR6000, HACH) in a quartz cell (light path 10 mm) at 25 °C with a PTC-348WI temperature controller. Fluorescence spectra were recorded on Thermo Scientific Lumina Fluorescence Spectrometer (THERMO-LUMINA) in a quartz cell (light path 10 mm) at 25 °C. Dynamic light scattering (DLS) was recorded on BI-200SM (Brookhaven Company) at 25 °C. CytoFLXE S Flow Cytometer (Beckman Coulter) was used to flow cytometry analysis experiments. Confocal laser scanning microscopy (Leica Stellaris 5 with Dmi8, Leica Dmi8 (Color camera)) was used in cell imaging experiments. Multimode microplate reader (FlexStation3) was used to evaluate the cck-8 results.

**DLS Experiment:**

The solvent was deionized water + 1‰ DMSO, filtered through a 0.22 μm filter. The sample was a clear, homogeneous dispersion with a concentration of 5 μM. The prepared sample solution was slowly and steadily poured into a clean sample cell, avoiding the formation of air bubbles. Three parallel samples were prepared for each group of samples, and each sample was then tested.

**SEM Sample Preparation:**

The solvent was deionized water + 1‰ DMSO, filtered through a 0.22 μm filter. The sample was a clear, homogeneous dispersion with a concentration of 5 μM. 5 μL of the prepared sample was dropped onto the smooth surface of a silicon wafer and then allowed to air dry overnight. Using an ion sputtering instrument, a platinum target was bombarded in an argon atmosphere to ensure uniform sputtering onto the sample surface, improving the sample's conductivity. The sample was then firmly attached to a metal sample post using conductive adhesive. Three parallel samples were prepared for each group, and each sample was tested.

**Singlet oxygen generation in the solution**

A sample solution was prepared with a concentration of 25.0 μM in 2.5% DMSO in PBS. 9,10-Anthracenediyl-bis(methylene)dimalonic acid (ABDA), a single oxygen probe, was used at a concentration of 50.0 μM. Under 650 nm laser irradiation, ABDA was gradually consumed by photochemically generated singlet oxygen (¹O₂). The decrease in the UV-Vis absorbance of ABDA was measured by UV-Vis spectroscopy.

**Cell viability assay**

To investigate the toxicity of intracellular polymerization to the B16 cells, the cells were cultured in 96-well plates in 1640 medium containing 10% FBS for 24 h, and then the corresponding samples were added into the wells, the cells were further cultured for 12 h. Then, the cell growth was calculated by CCK-8 assay.

**Animal Ethical Statement**

All animal experiments were approved by the Animal Ethics Committee, China Technology Industry Holdings (Shenzhen) Co., Ltd (20220091).

**Inhibition of tumor growth experiments**

To examine the in vivo anticancer ability, 6-week-old female C57BL/6 mice were subcutaneously injected 10^6^ B16 cells into the right hind leg to establish a subcutaneous tumor model. After injection for 8 days, the tumor burden mice were blindly and randomly separated into five groups (n = 5) and injected with PBS (100 μL), a-PD-L1 (80 μg/mL, 100 μL), TCPP (25.0 μM, 100 μL), TCPP-Tyr (25.0 μM, 100 μL), TCPP-Tyr (25.0 μM, 100 μL), and TCPP-Tyr+a-PD-L1 (([TCPP-Tyr] = 25.0 μM, [a-PD-L1] = 80 μg/mL, 100 μL)) (once per 3 days), respectively. On the second day after the drug was injected, 650 nm laser was used to irradiate the tumor site for 5 min (a group injected with TCPP, a group injected with TCPP-Tyr and a group injected with TCPP-Tyr+a-PD-L1). Moreover, after 14 days, heart, liver, spleen, liver, kidney and tumor were collected, fixed by 4% formaldehyde solution, embedded with paraffin and sectioned into slices and observed.

**Bone marrow-derived dendritic cells (BMDC) culture**

1. Harvest femur and tibia from C57BL/6 mice postmortem.
2. Dip bones in 70% ethanol (15 s), then wash with sterile PBS.
3. Cut the ends of the femur and tibia with sterile scissors. Rinse the bones with PBS containing 1% BSA and insert a syringe into the bones and collect the bone marrow into a sterile centrifuge tube. Rinse the bones repeatedly until all the bone marrow is collected.
4. Centrifuge the cells at 200 g for 5 minutes and discard the supernatant.
5. Lyse red blood cells (5 min), then neutralize with PBS and centrifuge (200 g, 5 min).
6. Resuspend the cell pellet in RPMI-1640 complete medium supplemented with 50 nM β-mercaptoethanol and 20 ng/mL GM-CSF, and cultured at 37 °C, 5% CO_2_.
7. Medium refresh:

Day 3: add 1 mL of fresh medium containing GM-CSF/β-mercaptoethanol.

Day 6: replace half of the medium with fresh medium containing GM-CSF/β-mercaptoethanol.

Day 7: Harvest: harvest cells.

**Bone marrow-derived macrophages (BMDM) culture**

1. Harvest femur and tibia from C57BL/6 mice postmortem.
2. Dip bones in 70% ethanol (15 s), then wash with sterile PBS.
3. Cut the ends of the femur and tibia with sterile scissors. Rinse the bones with PBS containing 1% BSA and insert a syringe into the bones and collect the bone marrow into a sterile centrifuge tube. Rinse the bones repeatedly until all the bone marrow is collected.
4. Centrifuge the cells at 200 g for 5 min and discard the supernatant.
5. Lyse red blood cells (5 min), then neutralize with PBS and centrifuge (200 g, 5 min).
6. Resuspend the cell pellet in RPMI-1640 complete medium supplemented with 20 ng/mL M-CSF, and cultured at 37 °C, 5% CO_2_.
7. Medium refresh:

Day 3: add 1 mL of fresh medium containing M-CSF.

Day 6: replace half of the medium with fresh medium containing M-CSF.

Day 7: Harvest: harvest cells.

**CD8^+^ T cells culture**

Isolation of CD8^+^ T cells (Kit: Invitrogen, Cat# 11417D)

1. Harvest the spleen from C57BL/6 mice, homogenize, and filter through a cell strainer. Centrifuge at 400 g for 5 min.
2. Lyse red blood cells (5 min), then neutralize with PBS and centrifuge (400 g, 5 min). Discard supernatant and wash cells twice with PBS.
3. Resuspend cells in 500 µL Labeling Buffer and add 50 µL antibody. Incubate at 4°C for 20 min.
4. Add 10 mL Labeling Buffer to neutralize, then centrifuge and discard supernatant.
5. Resuspend cells in 4 mL Labeling Buffer, add 500 µL magnetic beads, and incubate on a rotator at RT for 20 min.
6. Add 5 mL Labeling Buffer, mix gently, and place the tube on a magnetic stand.
7. Transfer the supernatant, centrifuge (400 g, 5 min), and resuspend in PBS.

T Cell Culture

1. Resuspend 1 × 10⁶ cells/mL in 1 mL RPMI-1640 medium and seed in a 24-well plate. Add IL-2 (10 ng/mL) and T cell activator beads (1 µL, Gibco™ Cat# 11453D). Culture for 3 days to generate effector T cells (replace medium containing IL-2 daily).
2. After 72 h of activation, replace IL-2 with L-15 (10 ng/mL) to promote memory T cell differentiation.

**Disperse tumor tissue into single cells**

1. Prepare digestive enzymes: 10 mg collagenase (Ⅱ), 1 mg hyaluronidase and 200 U DNase I were dissolved in 10 mL PBS and then filtered with a 0.22 um filter membrane.

2. Cut the tumor tissue into blocks with a volume of 0.5 - 1 mm^3^, add 1 mL digestive enzyme, gently blow and place at 37 °C for digestion for 2 h.

3. After digestion, filter with a 40 μm mesh, wash with 5 mL PBS and centrifuge (300 g, 5 min) and discard the supernatant.

4. After lysing the red blood cells with red blood cell lysis buffer, wash with 5 mL PBS, centrifuge (300 g, 5 min), discard the supernatant, and resuspend the precipitate with 1mL PBS to obtain a single cell suspension of tumor tissue.

**Gating Strategies for Flow Cytometry**

1. Fragmentation exclusion based on FSC-A and SSC-A.

2. Removal of adherent cells using FSC-H and FSC-A.

3. Gating of live cells using dead cell dye (PI) (tissue samples).

4. Identification of target cell populations based on specific surface markers.

**The imaging parameters for IVIS**

200 μL (15 mg mL^-1^) of sodium fluorescein was injected intraperitoneally into the mice before in vivo imaging.

Mode: luminescence

Exposure time: 1 s

Binning: 4

Emission: 570 nm

**Bilateral tumor experiment**

6-week-old female C57BL/6 mice were subcutaneously injected 10^6^ B16 cells into the left hind leg, 2×10^6^ B16 cells into the right hind leg to establish a bilateral subcutaneous tumor model. After injection for 8 days, the tumor burden mice were blindly and randomly separated into three groups (n = 5), treating only the right-sided tumor. PBS (100 μL), a-PD-L1 (80 μg/mL, 100 μL), TCPP (25.0 μM, 100 μL), and TCPP-Tyr (25.0 μM, 100 μL) (once per 3 days), respectively. On the second day after the drug was injected, 650 nm laser was used to irradiate the tumor site for 5 min, for a total of three treatments. Tumor size, body weight, and survival were assessed in the mice during the 14-day treatment period. After 14 days, tumors were collected and observed.

**Safety evaluation**

At experimental endpoint of anti-tumor treatment, organs (heart, liver, spleen, lung and kidney) and tumors collected from all groups for pathologic analysis on potential side effects and whole blood and serum were collected from PBS and TCPP-Tyr+a-PD-L1 (+) groups. The levels of serum ALT, AST, TP, CREA, and BUN in all treated mice were quantified by biochemical analysis. In addition, histological analysis was conducted on heart, liver, spleen, lung and kidney. The content of WBC, RBC, HGB, HCT, MCV, MCH, RDW, MPV, Lym# and Lym% was measured.

**Figure S1.** Synthetic route of TCPP-Tyr.

**Synthesis of TCPP-Tyr**

Meso-tetra(4-carboxyphenyl)porphine (50 mg, 63 μmol), methyl L-tyrosinate (98.7 mg, 505.6 μmol) and benzotriazol-1-yl-oxytripyrrolidinophosphonium hexafluorophosphate (PyBop) (164.5 mg, 316.1 μmol) were mixed in 20 mL DMSO. The mixture was then stirred at room temperature for 0.5 h. When the solution turned clear, Triethylamine (TEA) (40 μL) was added, and the reaction solution was stirred at room temperature for another 12 h. A purple solid (TCPP-Tyr) was obtained in a yield of 25% after purification through silica gel column chromatography by using an eluent containing CH_2_Cl_2_ and methanol (v/v, 25/1).

**Figure S2.** ^1^H NMR spectrum (600 MHz, DMSO-*d*_6_, 25 °C) of TCPP-Tyr.

**Figure S3.** ^13^C NMR spectrum (150 MHz, DMSO-*d*_6_, 25 °C) of TCPP-Tyr.


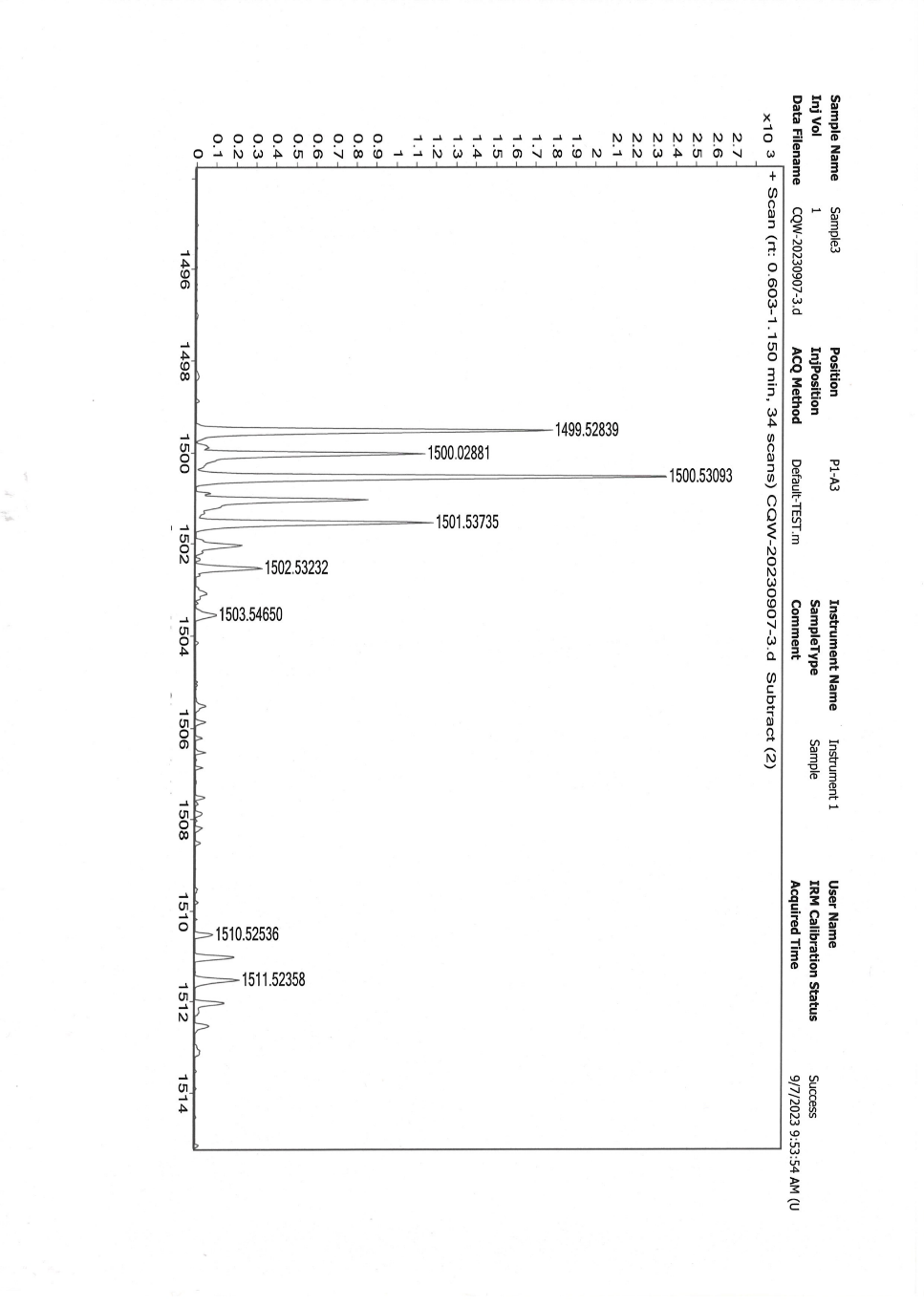

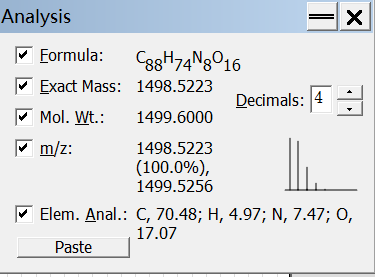


**Figure S4.** HRMS spectrum of TCPP-Tyr.


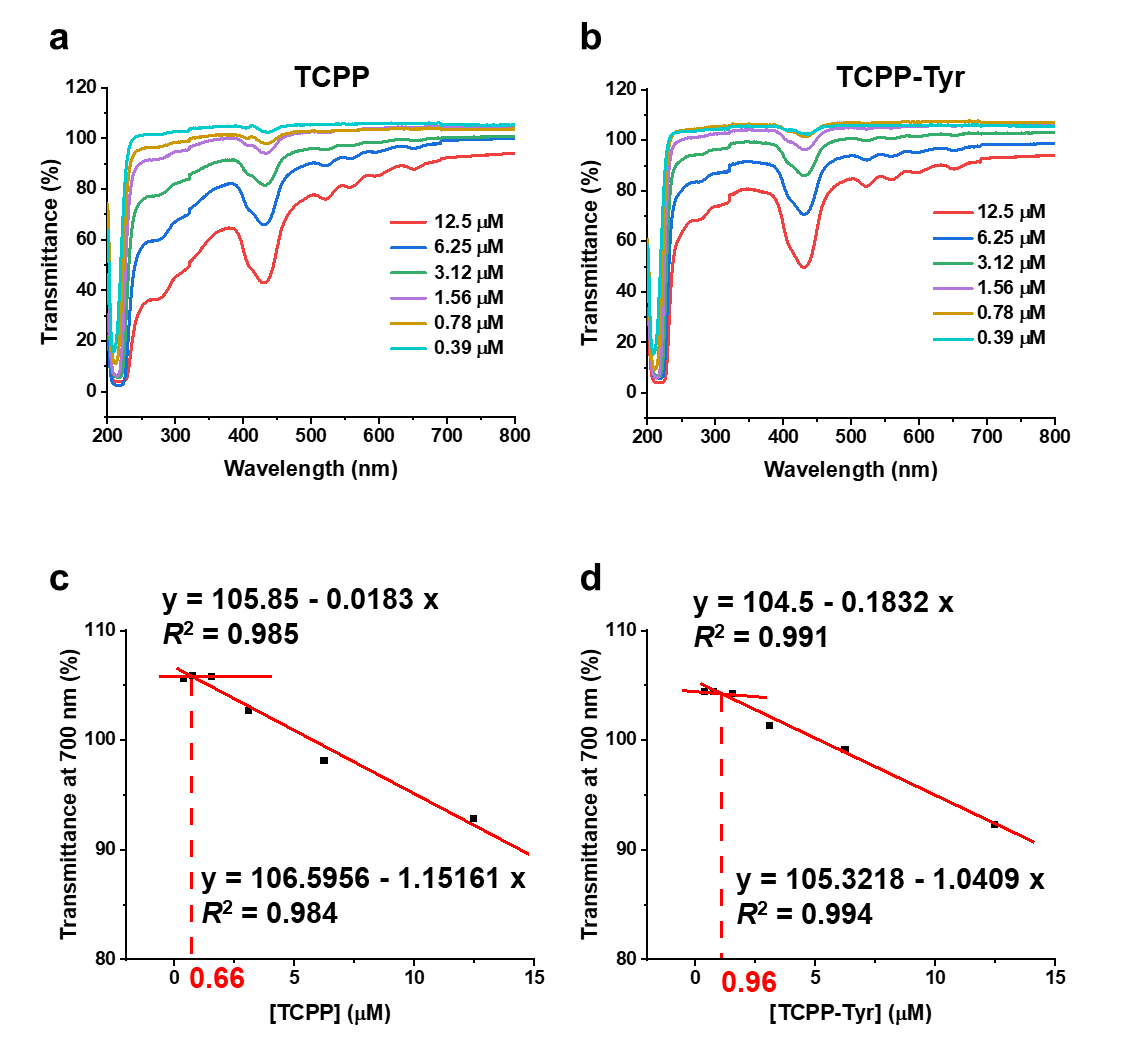


**Figure S5.** Optical transmittance of (a) TCPP and (b) TCPP-Tyr at different concentrations in PBS (pH = 7.2) at 25 ℃. Dependence of the optical transmittance at 700 nm on (c) TCPP and (d) TCPP-Tyr concentration in PBS (pH = 7.2) at 25 ℃.

**Figure S6.** FT-IR spectra of TCPP and Tyr.


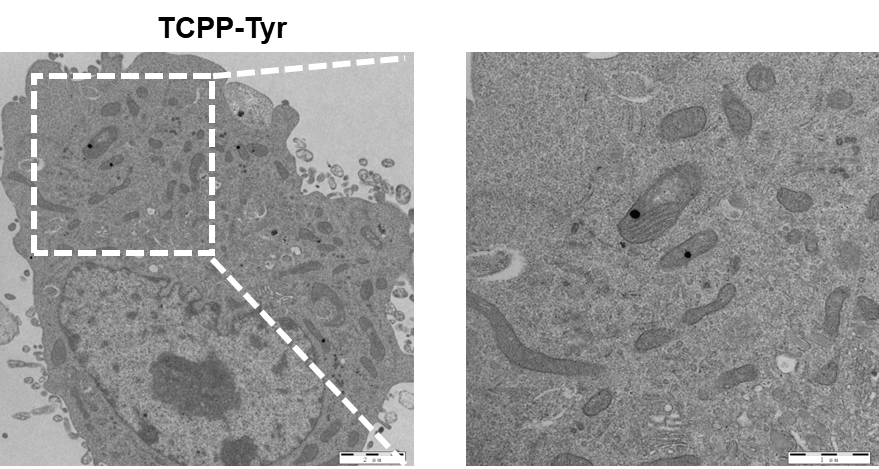


**Figure S7.** Bio-TEM images of HaCat cells incubated with TCPP-Tyr (12.5 μM) for 12 h.


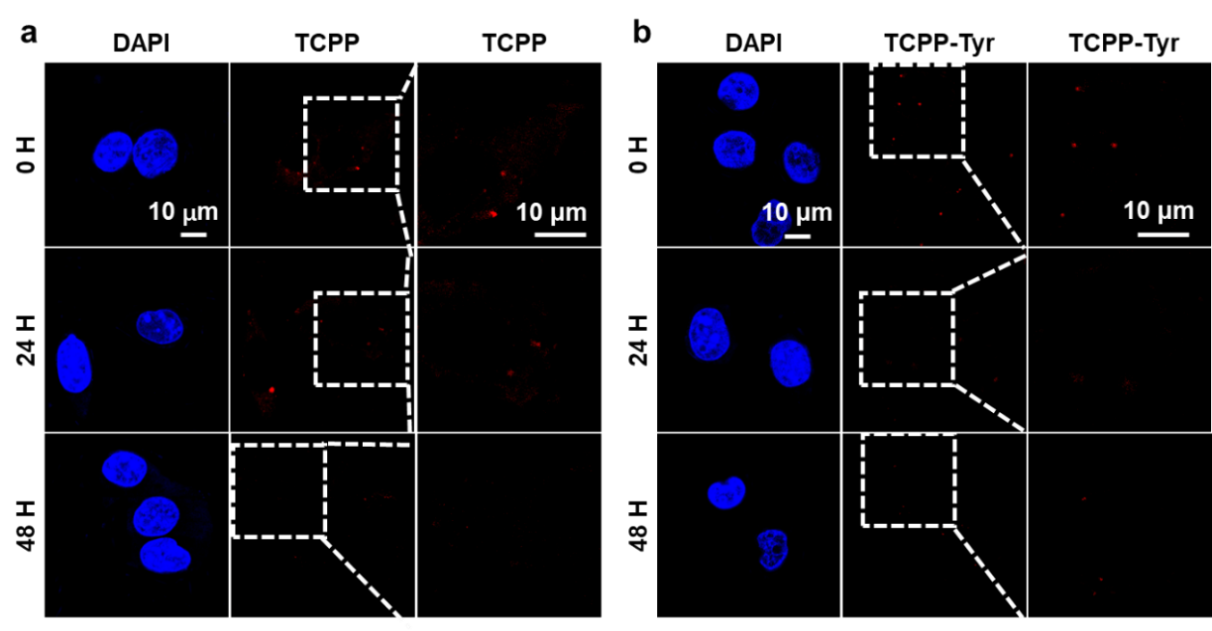


**Figure S8.** CLSM images of (a) B16 cells incubated with TCPP (12.5 μM), (b) HaCat cells incubated with TCPP-Tyr (12.5 μM) for 12 h, then the cells were cultured with fresh medium for different time.


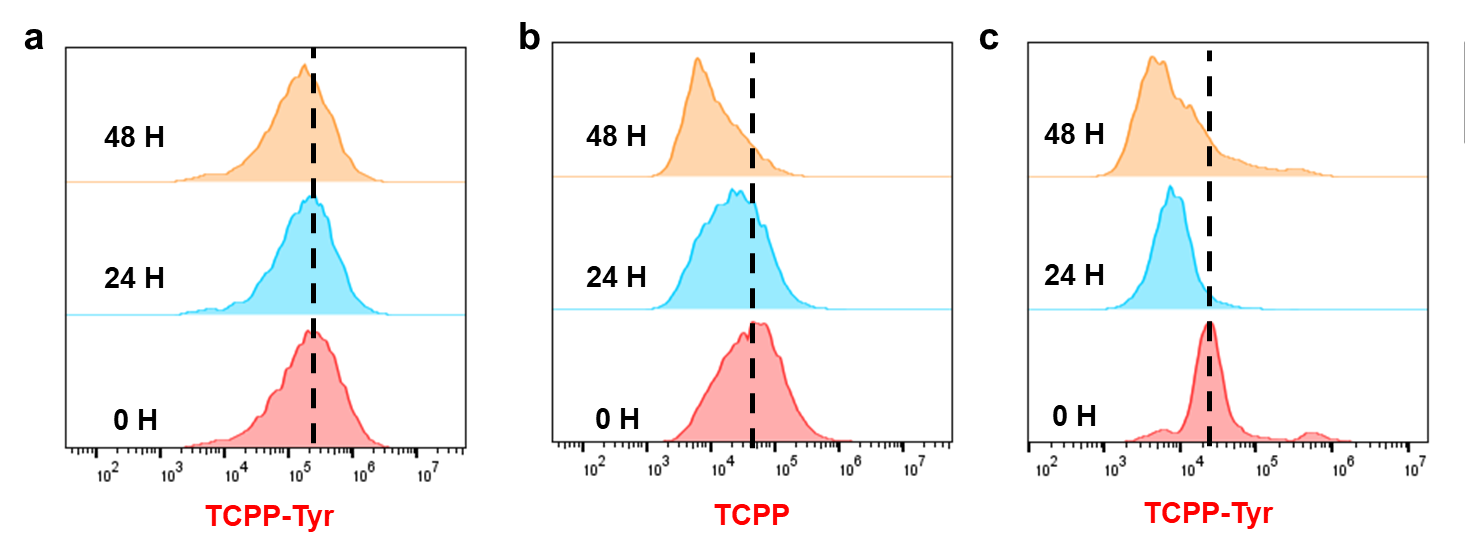


**Figure S9.** Representative flow cytometry results of (a) B16 cells incubated with TCPP-Tyr (12.5 μM), (b) B16 cells incubated with TCPP (12.5 μM), (c) HaCat cells incubated with TCPP-Tyr (12.5 μM) for 12 h, then the cells were cultured with fresh medium for different time.


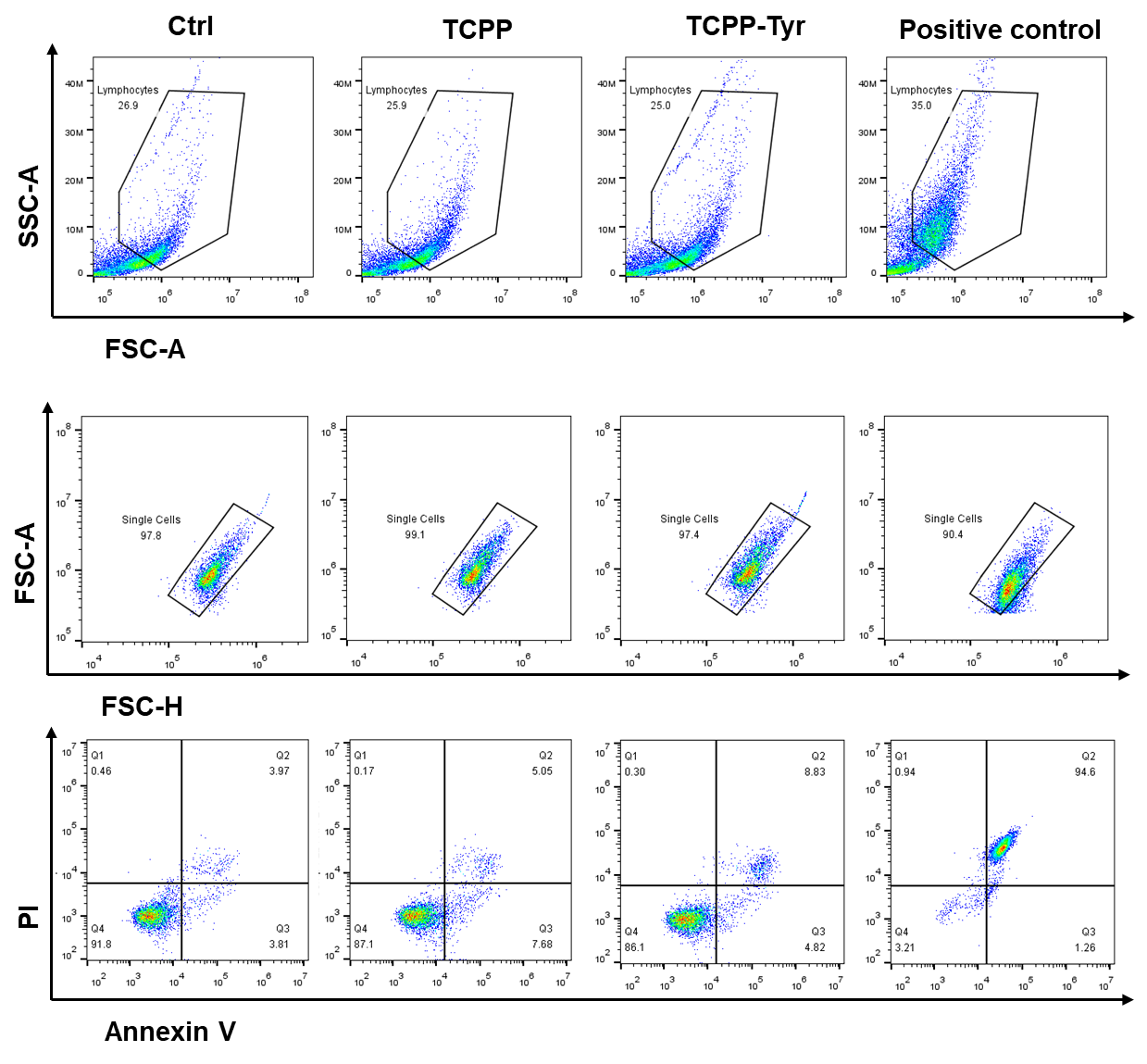


**Figure S10.** Annexin V-FITC/PI co-staining flow cytometry results of B16 cells incubated with different treatments for 12 h.


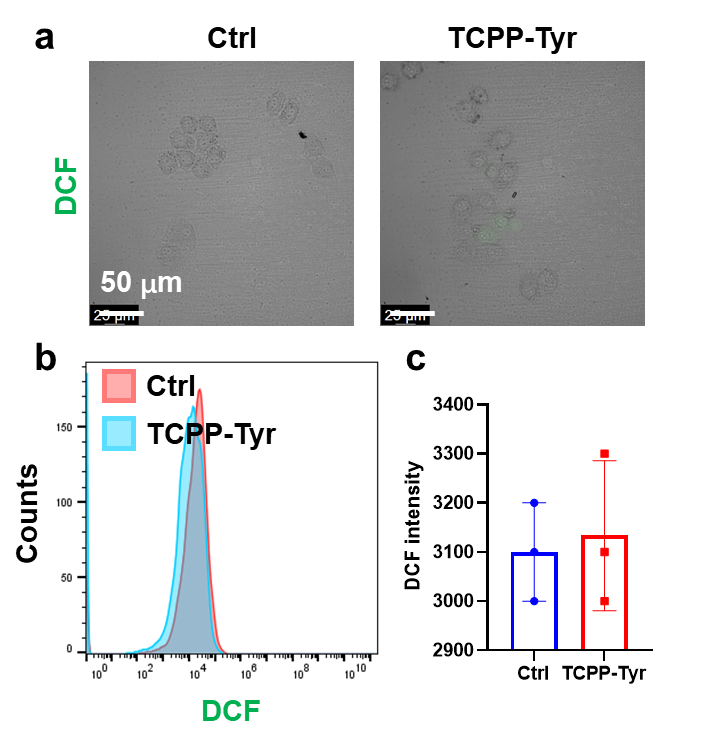


**Figure S11.** (a) CLSM images of ROS generation in HaCat cells after incubation without or with TCPP-Tyr in PBS for 12 h, as indicated by the green fluorescence of DCF that was oxidized from H_2_DCFDA by ROS. (b) Representative flow cytometry results of ROS in HaCat cells after incubation without or with TCPP-Tyr in PBS for 12 h. (c) Quantified content of ROS in HaCat cells. [TCPP-Tyr] = 12.5 μM.


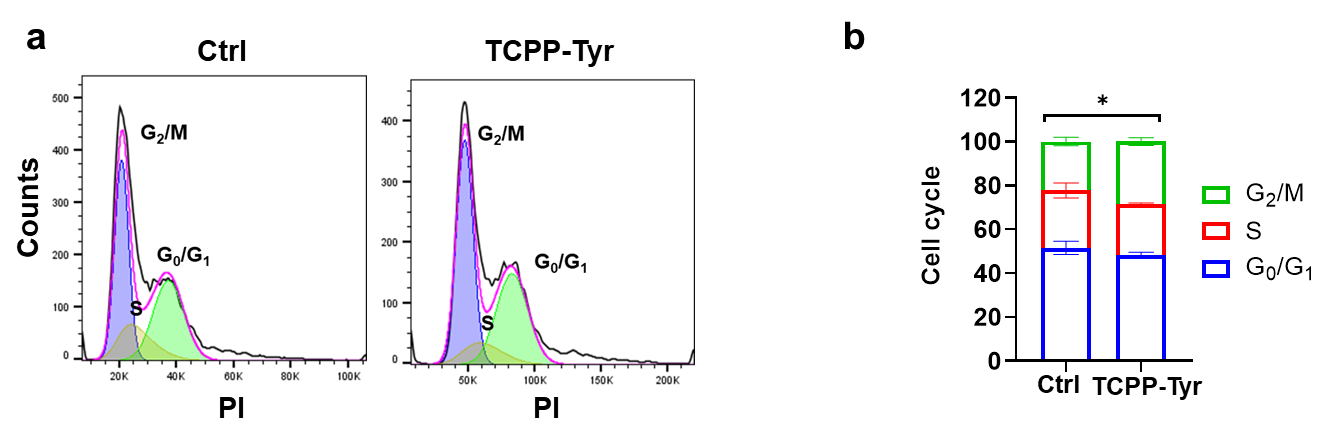


**Figure S12.** Cell cycle arrest caused by intracellular self-assembly. Treated cells were fixed with 70% ethanol overnight, treated with RNase A, stained by PI, and analyzed by flow cytometry. **P* ≤ 0.05 by students’ t test was applied to annotate statistical significance.


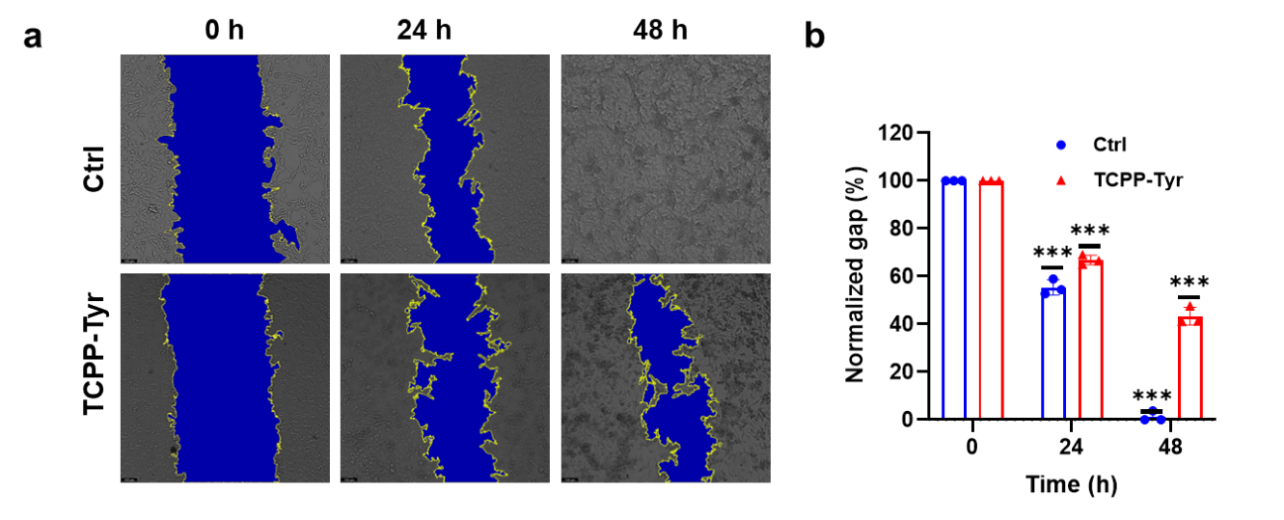


**Figure S13.** (a) Cell migration was determined by a wound-healing assay of B16 cell monolayers. (b) Normalized gaps versus time (calculated as the ratio of the remaining gap area at a given time point and at t = 0). ****P* ≤ 0.001 by one-way ANOVA was applied to annotate statistical significance.


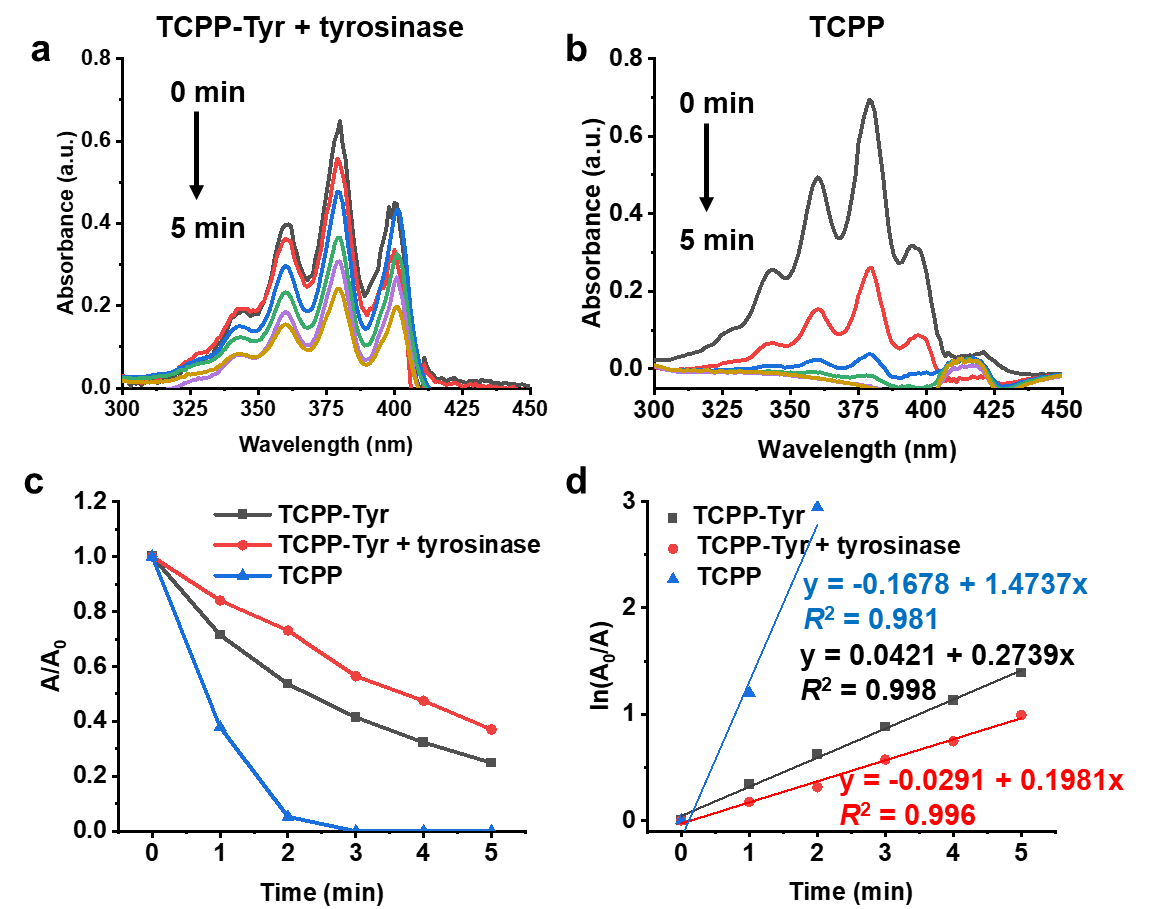


**Figure S14**. UV-vis spectra of ABDA in the presence of (a) TCPP-Tyr + tyrosinase, (b) TCPP under 650 nm light irradiation. The decreased UV-vis absorbance indicates that ABDA was gradually consumed by the photochemically generated ^1^O_2_. (c) Normalized UV-vis absorbance of ABDA at 380 nm after photodecomposition by ^1^O_2_ upon light irradiation at 650 nm. (d) Decomposition rate of ABDA with TCPP, TCPP-Tyr and TCPP-Tyr + tyrosinase. ([TCPP-Tyr] = [TCPP] = 25 μM, [ABDA] = 50 μM, [tyrosinase] = 1 mg/mL, 2.5% DMSO in PBS).


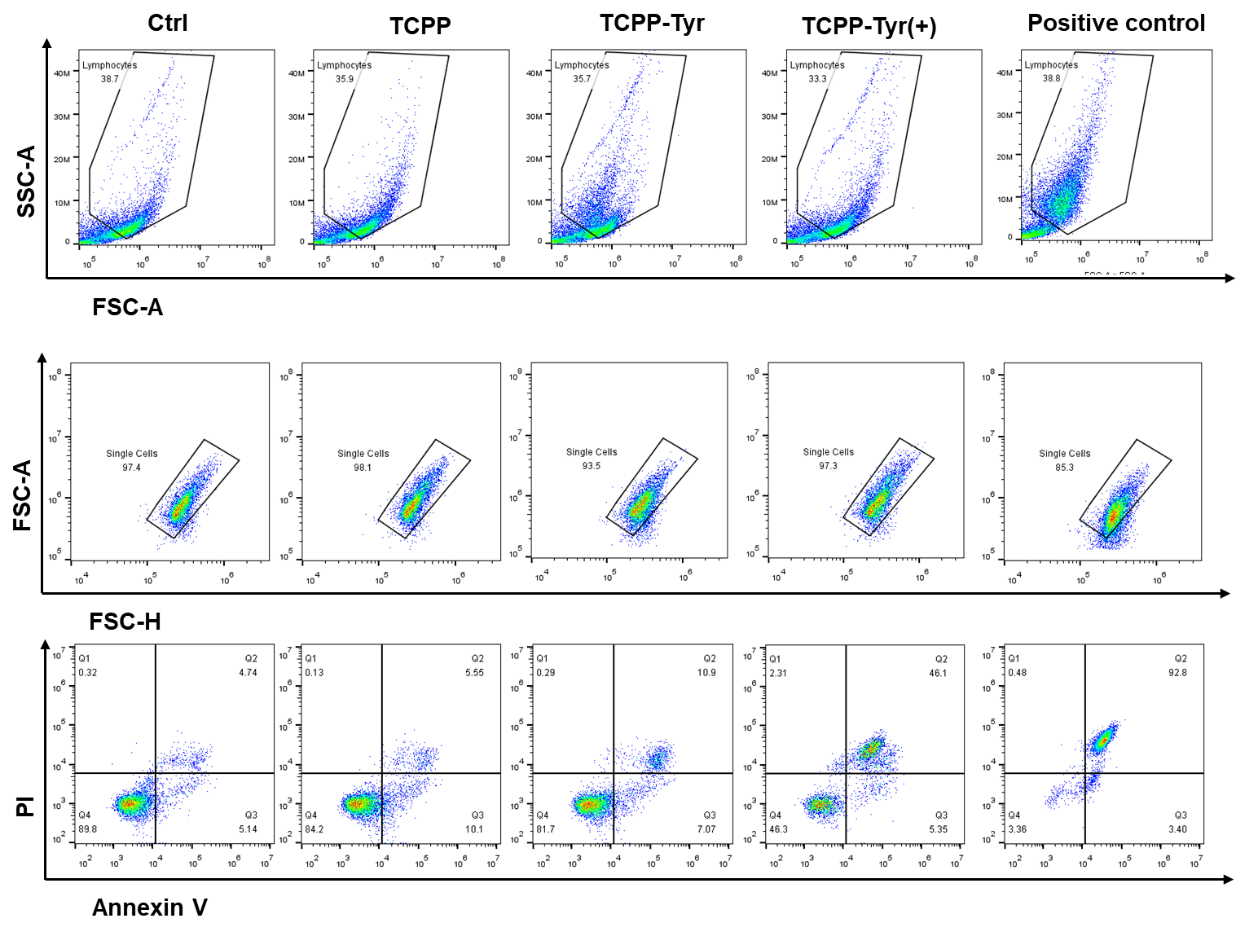


**Figure S15.** Annexin V-FITC/PI co-staining flow cytometry results of B16 cells incubated with different treatments for 12 h.


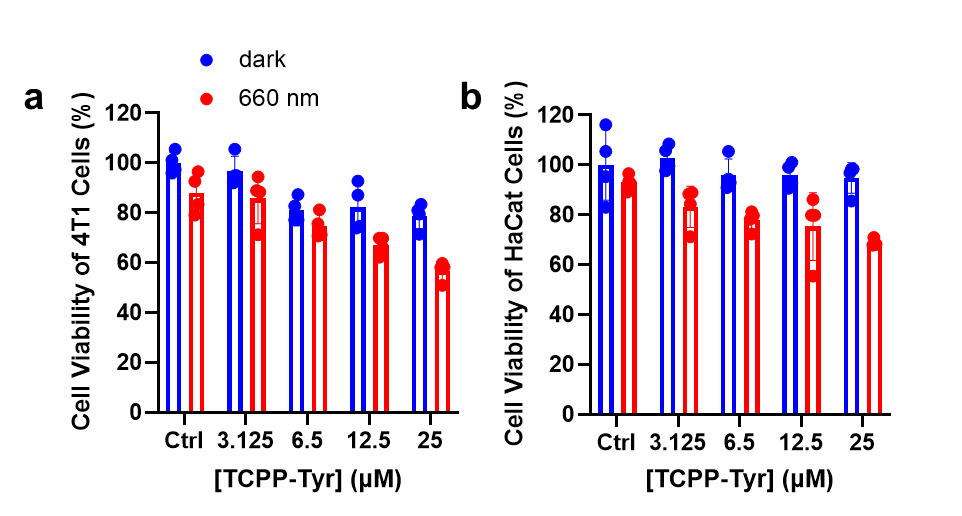


**Figure S16**. Cell viability of (a) 4T1 cells and (b) HaCat cells treated with TCPP-Tyr with or without light irradiation.


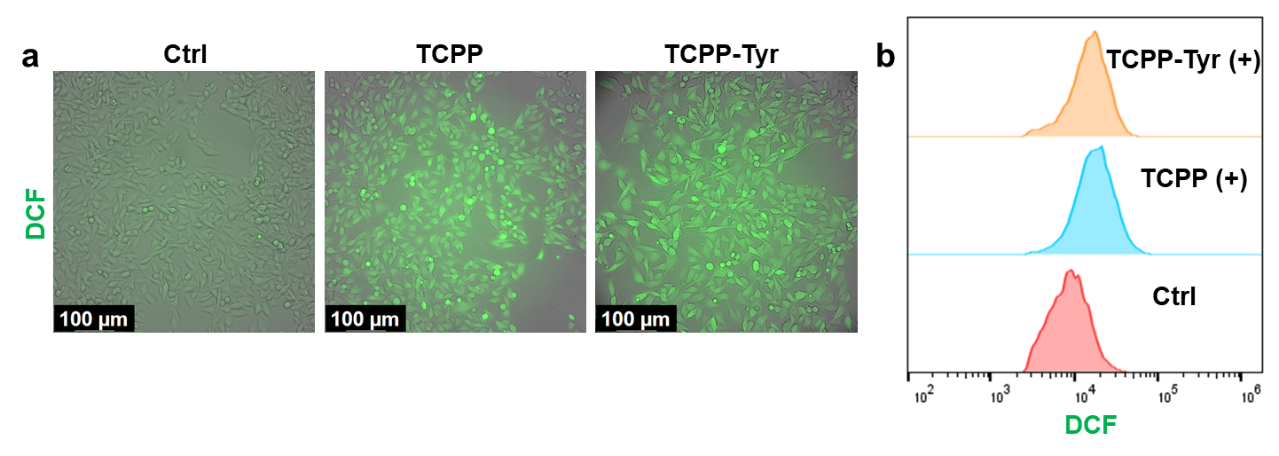


**Figure S17**. (a) Representative CLSM images of ROS generation in B16 cells and B16 cells treated with TCPP-Tyr or TCPP with light irradiation for 5 min (1.0 W/cm^2^). [TCPP-Tyr] = [TCPP] = 6.25 μM. (b) Representative ROS staining flow cytometry results of B16 cells and B16 cells treated with TCPP-Tyr or TCPP with light irradiation for 5 min (1.0 W/cm^2^). [TCPP-Tyr] = [TCPP] = 6.25 μM.


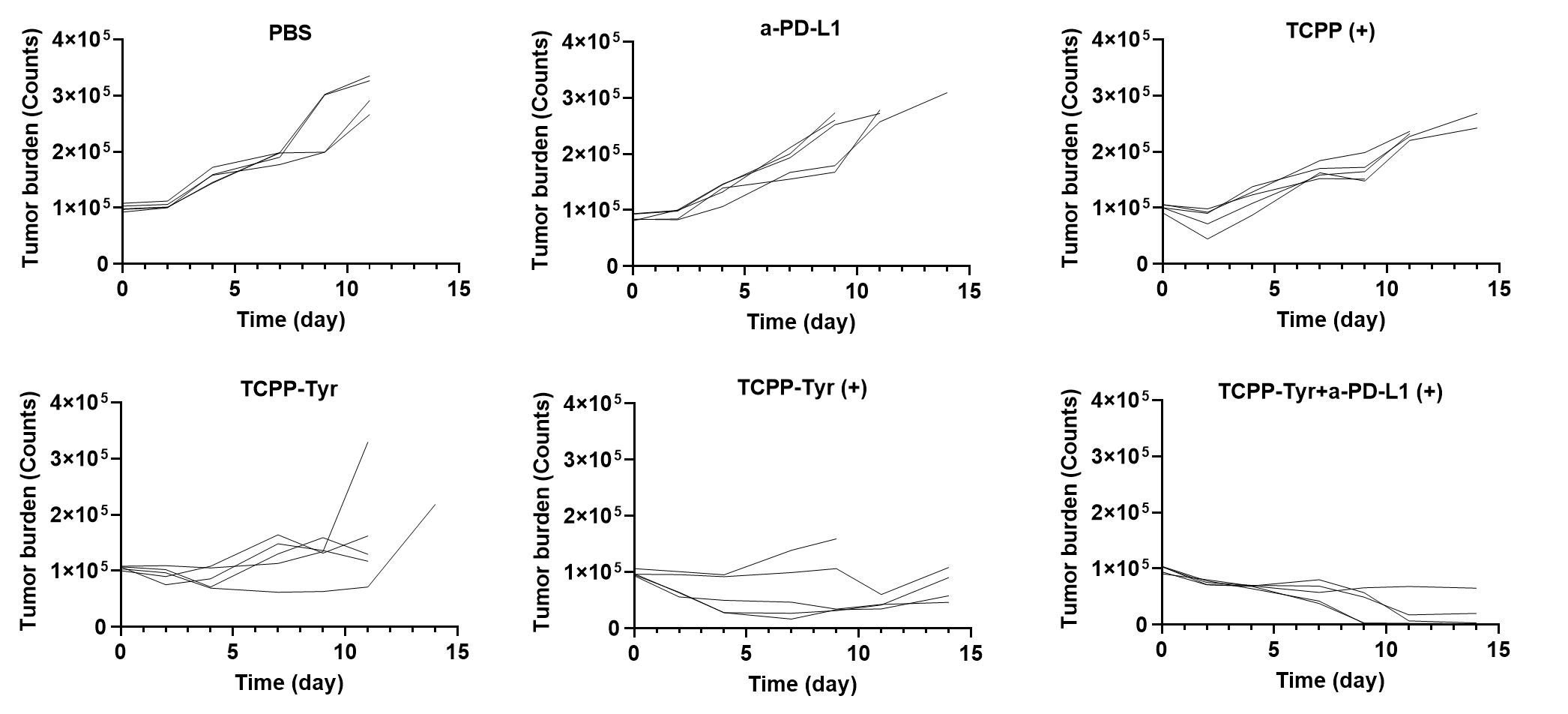


**Figure S18**. Graph of tumor burden (Counts) across time of PBS, a-PD-L1, TCPP (+), TCPP-Tyr, TCPP-Tyr (+) and TCPP-Tyr+a-PD-L1 (+) mice.


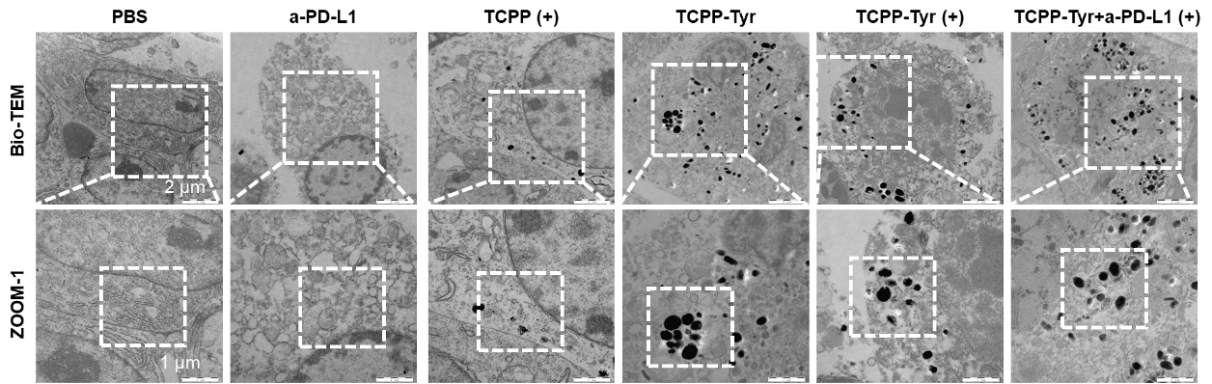


**Figure S19**. Bio-TEM images of tumor tissue of PBS, a-PD-L1, TCPP (+), TCPP-Tyr, TCPP-Tyr (+) and TCPP-Tyr+a-PD-L1 (+) mice.


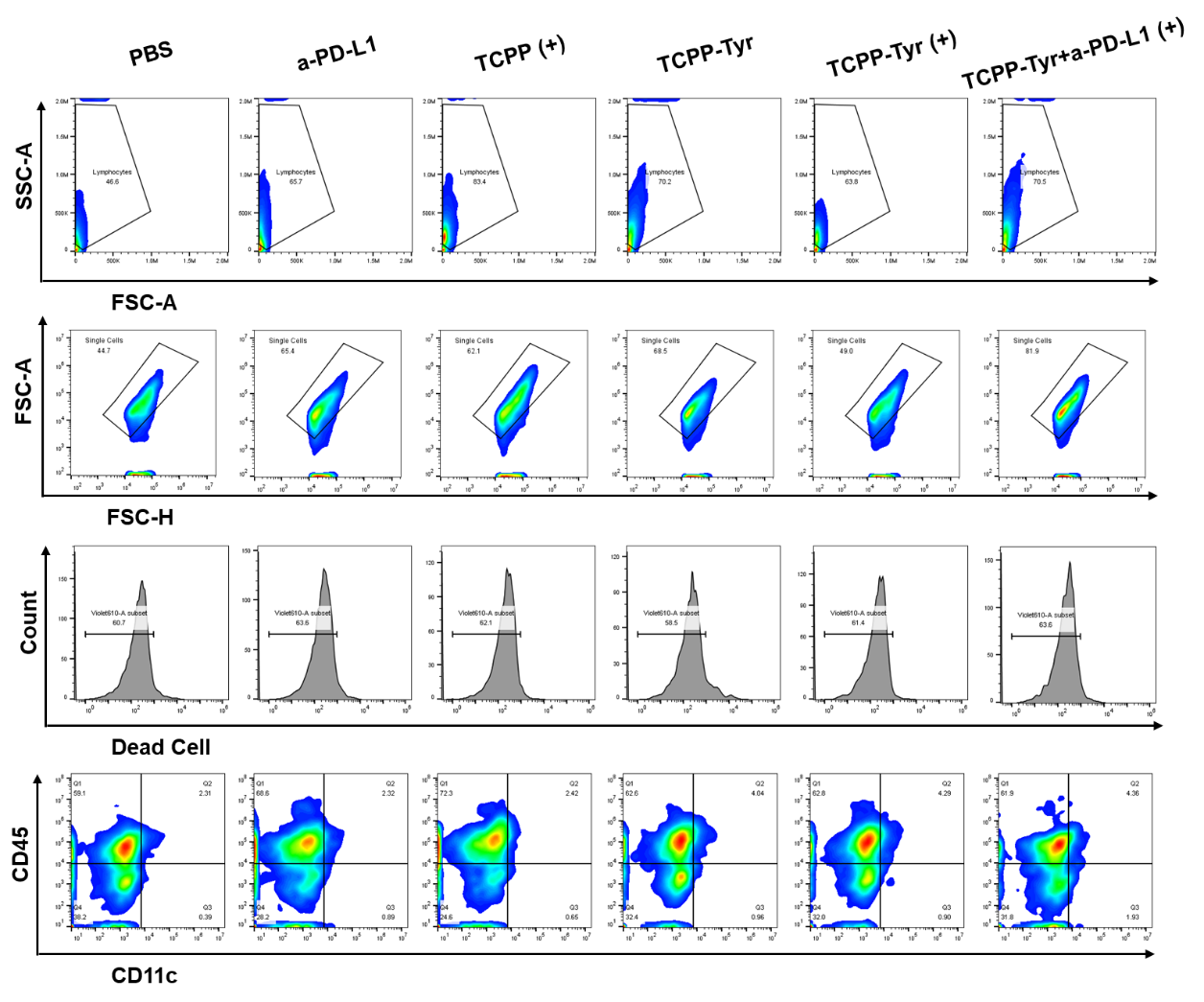


**Figure S20**. Representative flow cytometry analysis of the population of DC maturation (CD11c^+^CD45^+^) in tumors collected from B16 tumor-bearing mice after various treatments on day 14.


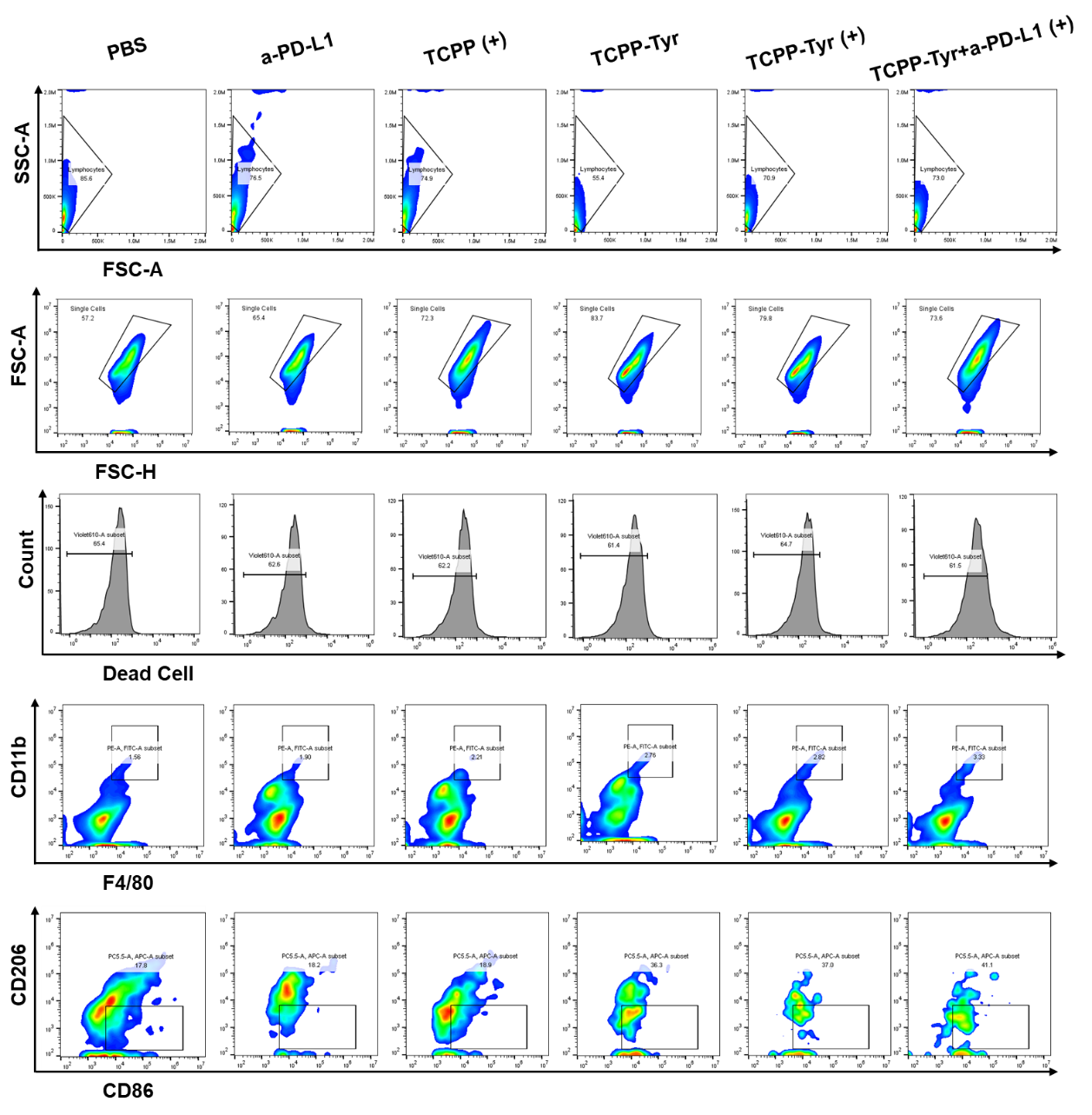


**Figure S21**. Representative flow cytometry analysis of the population of macrophage in tumors collected from B16 tumor-bearing mice after various treatments on day 14.


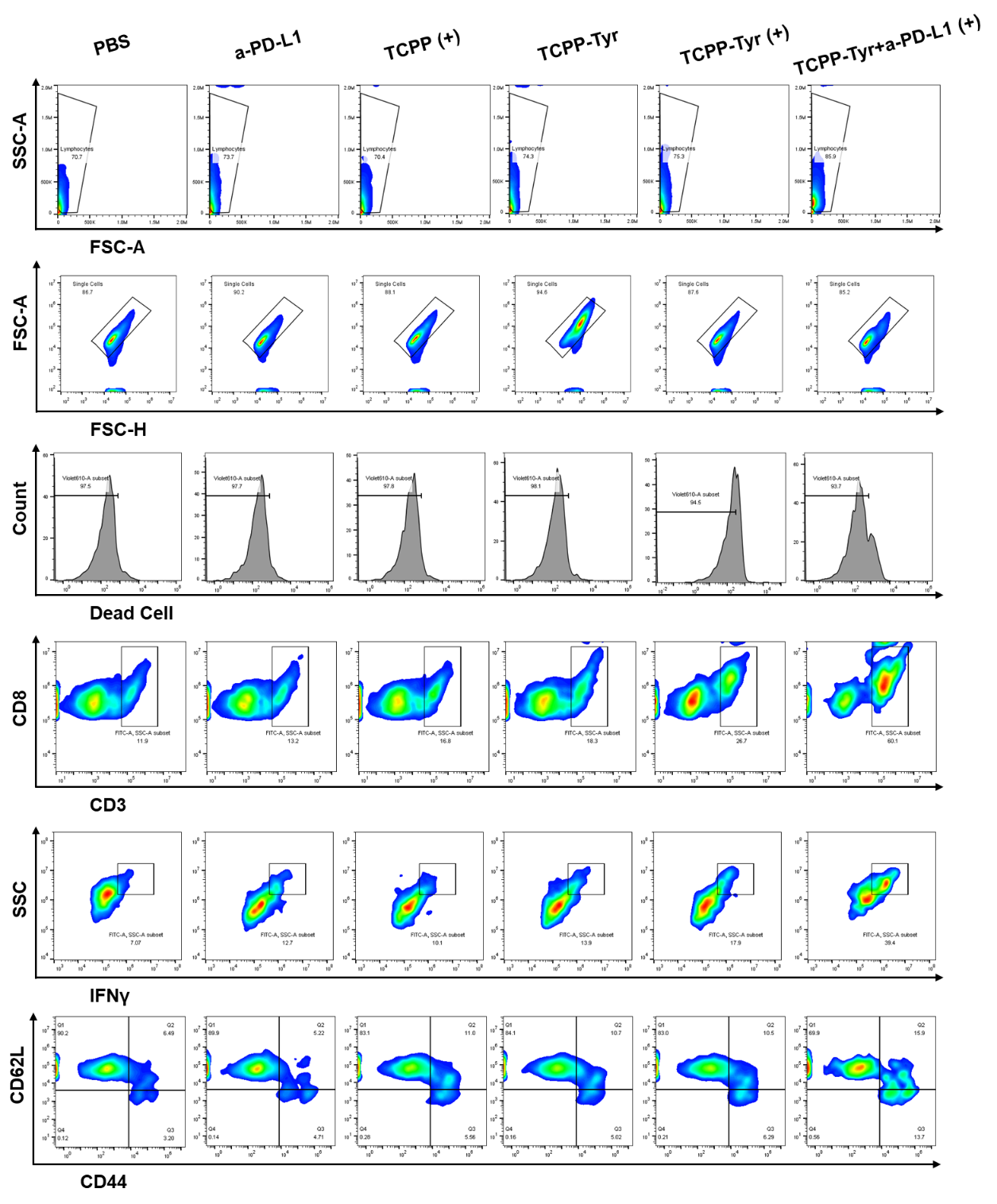


**Figure S22**. Quantitative data of the percentages of CD8^+^ T cells, IFN-γ^+^ cells and CD44^high^CD62L^low^ in tumors collected from B16 tumor-bearing mice after various treatments on day 14.


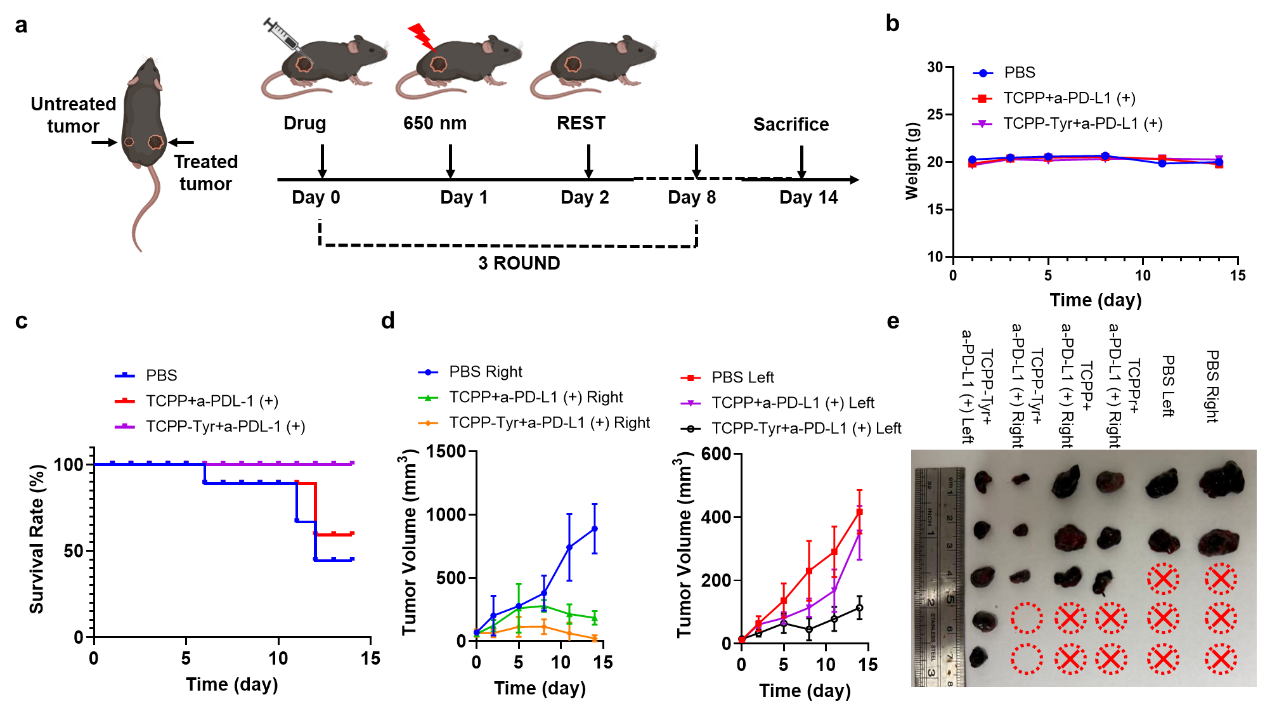


**Figure S23.** A bilateral tumor model was established in C57BL/6 mice by subcutaneous inoculation of tumors on both sides. Unilateral intratumoral administration was then performed. (a) Schematic illustration of antitumor experiment in mice. (b) Graph of body weight of mice across time of control and treated mice. (c) Survival rate curve of the mice during the 14-day treatment period. (d) Graph of tumor size across time of control and treated mice. (e) Representative photos of the excised tumors on day 14 after the various treatments. A hollow circle indicated that the tumor disappeared after treatment, while a circle with a cross indicated that the tumor volume of the mouse reached the threshold for euthanasia or that the mouse died naturally during treatment.


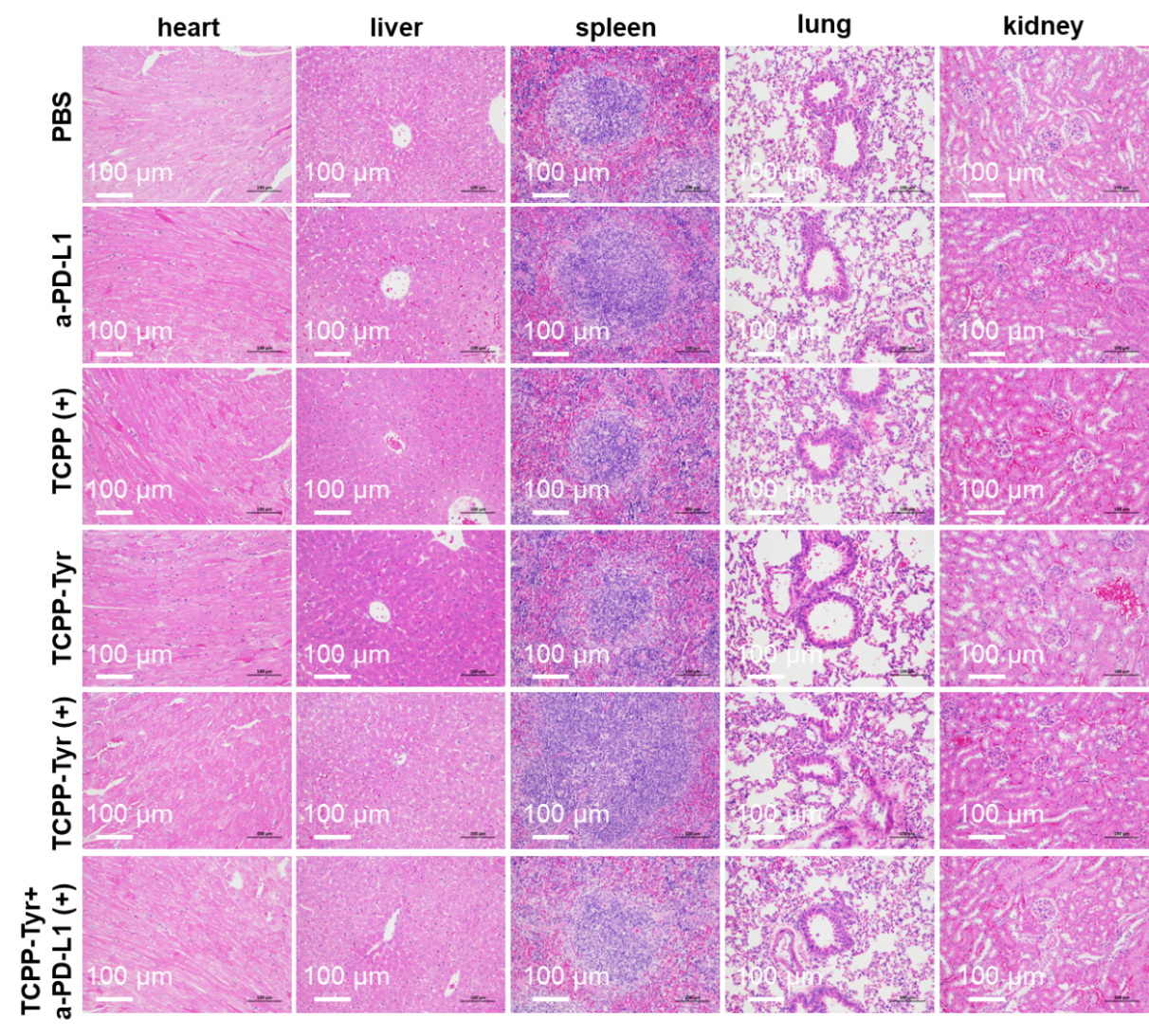


**Figure S24.** Tumor-bearing mice were randomly divided into six groups: PBS, a-PD-L1, TCPP (+), TCPP-Tyr, TCPP-Tyr (+) and TCPP-Tyr+a-PD-L1 (+) ([TCPP] = [TCPP-Tyr] = 25.0 μM, [a-PD-L1] = 80 μg/mL, 100 μL). After 3 treatments, the organs (heart, liver, spleen, lungs and kidneys) were collected for H&E staining.


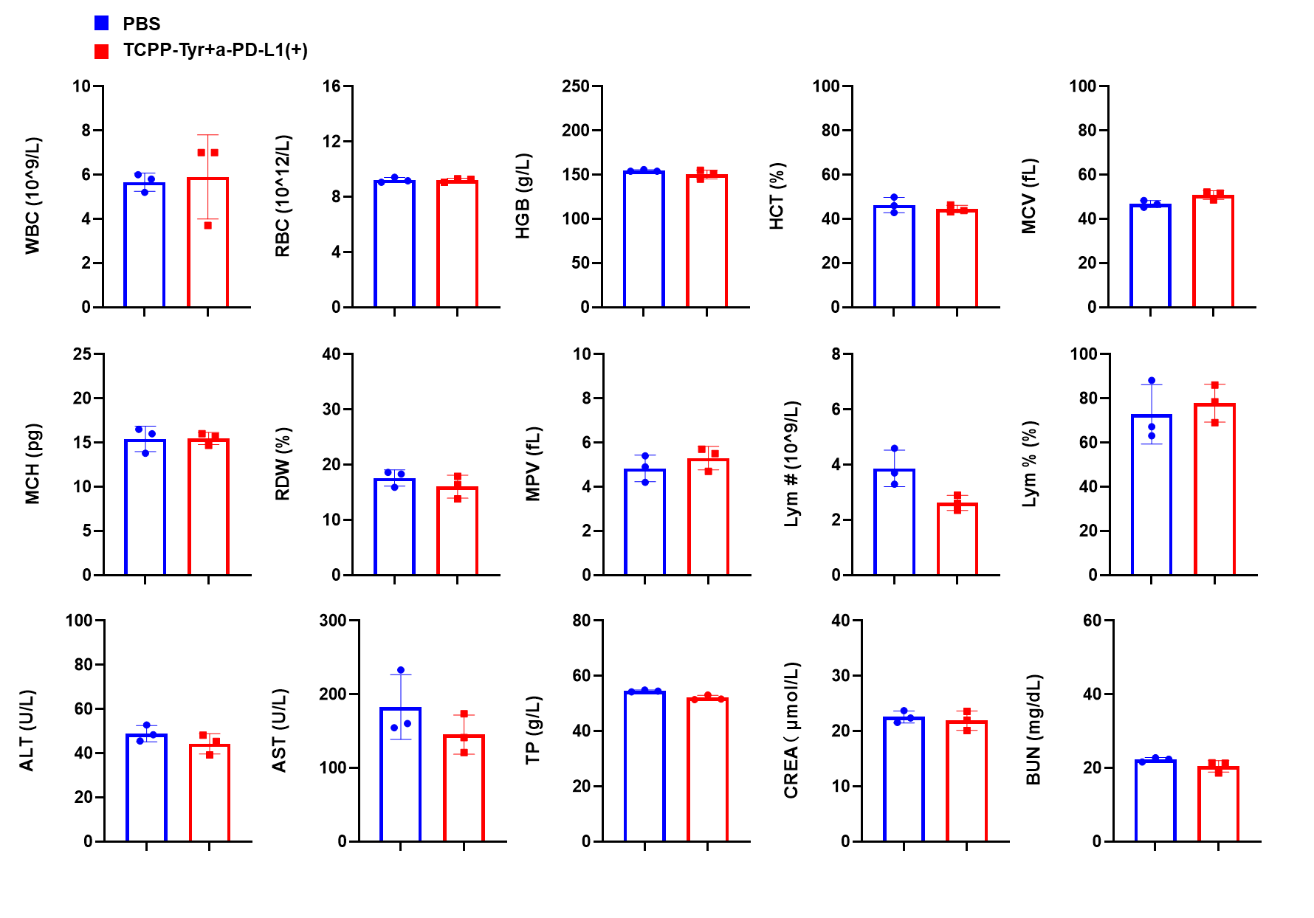


**Figure S25.** Tumor-bearing mice were randomly divided into two groups: PBS, and TCPP-Tyr+a-PD-L1 (+) ([TCPP-Tyr] = 25.0 μM, [a-PDL-1] = 80 μg/mL, 100 μL). After 3 treatments, whole blood from PBS group and TCPP-Tyr+a-PD-L1 (+) group were for studies of the routine blood tests; serum from PBS group and TCPP-Tyr+a-PD-L1 (+) group were for studies of typical indicators of liver and kidney function.
